# Understanding the role of oxylipins in *Cannabis* to enhance cannabinoid production

**DOI:** 10.3389/fpls.2025.1568548

**Published:** 2025-04-24

**Authors:** Gayathree I. Senevirathne, Anthony R. Gendall, Kim L. Johnson, Matthew T. Welling

**Affiliations:** ^1^ Australian Research Council Research Hub for Medicinal Agriculture, Department of Ecological Plant and Animal Sciences, School of Agriculture, Biomedicine and Environment, La Trobe University, Bundoora, VIC, Australia; ^2^ La Trobe Institute of Sustainable Agriculture and Food, Department of Ecological Plant and Animal Sciences, School of Agriculture, Biomedicine and Environment, La Trobe University, Bundoora, VIC, Australia; ^3^ Australian Research Council Research Hub for Protected Cropping, Department of Ecological Plant and Animal Sciences, School of Agriculture, Biomedicine and Environment, La Trobe University, Bundoora, VIC, Australia; ^4^ Australian Research Council Research Hub for Sustainable Crop Protection, Department of Ecological Plant and Animal Sciences, School of Agriculture, Biomedicine and Environment, La Trobe University, Bundoora, VIC, Australia

**Keywords:** glandular trichomes, green leaf volatiles, jasmonates, lipoxygenase, oxylipins, specialized metabolites

## Abstract

Phytocannabinoids are medically important specialized defense compounds that are sparsely distributed among plants, yet *Cannabis sativa* can synthesize unprecedented amounts of these compounds within highly specialized surface cell factories known as glandular trichomes. The control mechanisms that allow for this high level of productivity are poorly understood at the molecular level, although increasing evidence supports the role of oxylipin metabolism in phytocannabinoid production. Oxylipins are a large class of lipid-based oxygenated biological signaling molecules. Although some oxylipins are known to participate in plant defense, roles for the majority of the ca. 600 plant oxylipins are largely unknown. In this review, we examine oxylipin gene expression within glandular trichomes and identify key oxylipin genes that determine the fate of common lipid precursors. Mechanisms by which oxylipins may be interacting with phytocannabinoid metabolism, as well as specialized plant metabolism more broadly, are discussed and a model summarizing these contributions proposed.

## 1 Introduction

*Cannabis sativa* L. *(Cannabis)* is a chemically complex plant that has a repertoire of more than 500 diverse molecules that span several classes of specialized metabolites, including a large class of medically important isoprenylated resorcinol aliphatic polyketides known as phytocannabinoids (PCs). PCs serve as the active pharmaceutical ingredients in medicines used as adjunct therapies for drug-resistant epilepsies (Epidiolex) and multiple sclerosis (Sativex), as well as nausea, pain, and loss of appetite (Dronabinol) (Lichtman et al., 2018; Thiele et al., 2018). In plants, PCs play a critical role in defense responses, including protection from UV radiation, herbivorous insects, and microbial pathogens, but the exact role of PCs in *Cannabis* is poorly understood (Tanney et al., 2021). While many of the enzymes responsible for producing these metabolites have been elucidated, the control mechanisms governing their synthesis remain underexplored (Stout et al., 2012; Welling et al., 2023). Increasing evidence suggests that PC production may be mediated by oxylipins (Bailey, 2019; Booth et al., 2020; Burgel et al., 2020; Apicella et al., 2022; Garrido et al., 2022; Welling et al., 2023; **Table 1**).

**Table 1:** Effect of JAs on PC production and glandular trichome density in *Cannabis*.

*Cannabis* variety	MeJA/JA treatment	Effect on PC production/trichome density	Changes in gene expression	Reference
**Medicinal (Skunk)**	100 μM jasmonic acid/0.3 mM MeJA applied to cell cultures	No detectable level of Δ^9^-THC	No detectable change in *THCAS*	Flores-Sanchez et al., 2009
**Industrial hemp (Santhica)**	0.03, 0.1, 0.3, 1, and 3 mM jasmonic acid applied to 15-day old seedlings	Not reported	Higher expression of *LOX2* and *4-coumarate-CoA ligase-like 7*	Behr et al., 2018
**Industrial hemp (Cherry, Cherry Blossom, Canada)**	44.9 mg/L MeJA applied as foliar and roots as separate treatments for 7weeks old plants	A significant increase in CBDA (>2-fold) in foliar applications in all three varieties A decrease in Δ^9^-THC in and increase in total PC content	Not reported	Bailey, 2019
**Medicinal (White Tangy Haze)**	0, 100, 500, and 1000 μM total plant spray MeJA after 2 weeks of flower development	A significant increase inΔ^9^-THC in 1000 μM at week 1 (>0.1-fold) and week 4 (>0.07-fold)	Not reported	Apicella et al., 2022
**Medicinal (Beatriz)**	0, 0.1, 1.0, and 10 mM MeJA, 8 applications (once a week) after shifting to reproductive photoperiod	An increase in Δ^9^-THCA (>0.08-fold) and CBDA (>0.15-fold) in response to 0.1 mM A decrease in Δ^9^-THCA and CBDA in response to 10 mM	No significant change in the expression of *AOC-1*, *AOC-2*, *PT4*, *THCAS*, and *CBDAS*	Garrido et al., 2022
**Not reported**	150 μM MeJA applied to 4-week-old plants	Not reported	Significant increase in expression of *CsLOX1*, *CsLOX2*, *CsLOX5*, *CsLOX8*, *CsLOX10*, *CsLOX11*, *CsLOX12*, *CsLOX3*, *CsLOX17* 2 hours and CsLOX*1*, *CsLOX2*, *CsLOX5*, *CsLOX10*, *CsLOX11*, *CsLOX12*, CsLOX16, 24 hours post application	Fayaz et al., 2023
**Industrial hemp (MW6-15)**	0, 1, 7.5, and 15 mM MeJA, foliar spray on 14th days after transitioning into reproductive photoperiod, after initiation of terminal flowering and 7 and 14-days post first application	A significant increase (~1-fold) in total cannabinoids, Δ^9^-THCA, THCVA CBDA, CBDVA, and CBCA (> 1-fold) in response to 15 mM	*JMT* responsive to 7.5 and 15 mM MeJA in the leaf and inflorescence. *LOX-L* and *HPL* highly expressed in the leaf and inflorescence	Welling et al., 2023
**Hemp (Hot Blond)**	100, 200, and 400 μM, foliar spray on 0 and 21 days after transitioning to reproductive photoperiod	A significant increase in glandular trichome density in calyx, A significant increase in CBD (>0.1-fold in both leaves and inflorescence) and Δ^9^-THC (>0.3-fold in leaves, >0.1-fold in inflorescence) in response to 100 mM	Not reported	Hahm et al., 2024
**CANN97**	1 µM MeJA applied 5 times at 3-day intervals to 18-day old plants	A significant increase in leaf sessile glandular trichome density and non-glandular trichome density	Significant increase in the expression of *CsJAZ1, CsJAZ2, CsJAZ3, CsMYC1, CsMYC4, CsCOI1*	Huang et al., 2024
**High CBD**	100 μM jasmonic acid as foliar spray, for consecutive 8 weeks after shifting to flowering photoperiod	No significant effect on overall glandular trichome density in bracts A decrease in Δ^9^-THCA, and CBDA but significant increase (> 0.2-fold) in Δ^9^-THC and CBD after 7 and 8 weeks in flowering	Not reported	Oultram et al., 2024
**Not reported**	Adventitious roots incubated in 0, 50, 100, 150, and 200 μM MeJA for 2 days	A significant increase (>0.5-fold) in CBD in 200 µM	Not reported	Wang Y, et al., 2024

**Figure 1:**
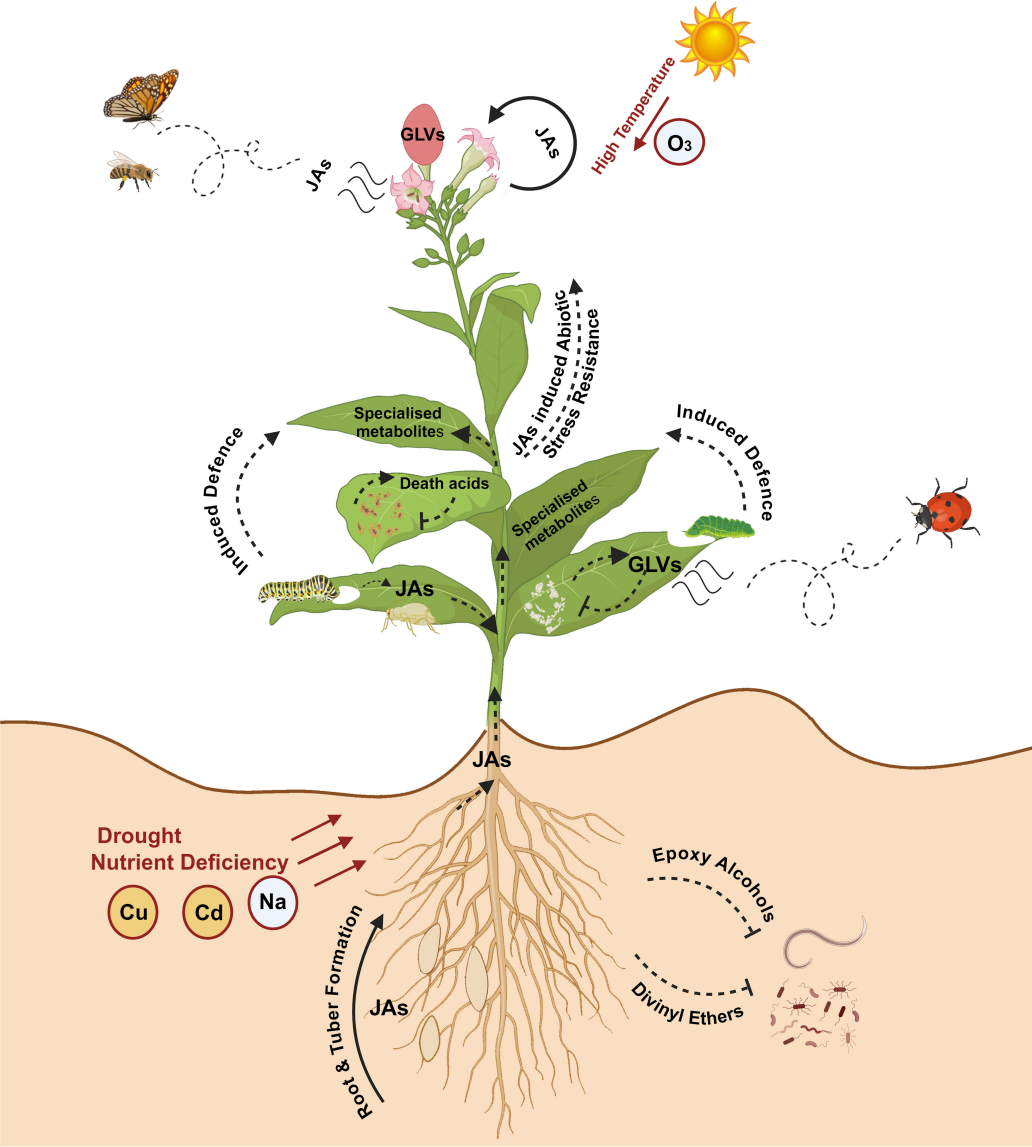
Role of oxylipins in plant growth, development, biotic and abiotic stress tolerance. Oxylipins play multifaceted roles in regulating plant growth, development, and responses to biotic and abiotic stresses. Jasmonates (JAs) mediate developmental processes, including lateral root formation, tuber formation, flowering, and trichome development. Several JAs are also known to attract pollinators and influence plant reproductive strategies. JAs activate defense genes in plants to protect against biotic and abiotic stress (nutrient deficiency, heavy metals [copper (Cu), cadmium (Cd)] and ozone (O_3_)). Green leaf volatiles (GLVs) contribute to fruit ripening and act as direct defense molecules against pathogenic microbes. GLVs also induce other defense responses, such as attracting natural predators and promoting the production of specialized metabolites. Oxylipins like epoxy alcohols and divinyl ethers serve as direct defense molecules against various pests and pathogens. Image created in BioRender.com.

Oxylipins are structurally and functionally diverse, lipid-based biological signaling molecules that participate in plant immunity (Blée, 2002; Christensen et al., 2016; Ameye et al., 2018; Wasternack and Feussner, 2018; Toporkova et al., 2024; **Figure 1**). Oxylipins play vital roles in regulating biological functions in plants including growth and development, innate and induced defense mechanisms as response to diverse environmental elicitors and as secondary messengers activating defense compounds (McGarry et al., 2024; Wang et al., 2024; Yokoyama et al., 2025). They are produced by the activity of lipoxygenases (LOXs) and various enzymes of the cytochrome P450 CYP74 family, such as allene oxide synthase (AOS) and hydroperoxide lyase (HPL) (Song and Brash, 1991; Howe et al., 2000; Wasternack and Feussner, 2018). Jasmonates (JAs), including jasmonate-isoleucine (JA-Ile), jasmonic acid, and its methylated form methyl jasmonate (MeJA) are oxylipin-derived phytohormones formed from the LOX-AOS cascade (Wasternack and Hause, 2013; Wasternack and Feussner, 2018; Ghorbel et al., 2021).

JAs induce specialized metabolism in diverse plant lineages, including *Solanum lycopersicum* (tomato) and *Lavandula* spp. (lavender), while also regulating the development of glandular trichomes and flowers where PCs are concentrated (Boughton et al., 2005; Chen et al., 2018; Dong et al., 2022a; Kianersi et al., 2022; Yan et al., 2022). More recently, JAs have been shown to enhance the levels of PCs, although the intricate mechanism(s) driving this increase in PC production is not fully understood (**Table 1**). There is also an emerging hypothesis that green leaf volatiles (GLVs) formed via the LOX-HPL branch of oxylipin metabolism may provide substrate for the synthesis of hexanoic acid, the polyketide starter unit for PC production, although only limited evidence has been reported supporting this interaction (Stout et al., 2012; Welling et al., 2023). There has been considerable gene expansion in the *LOX* family in *Cannabis* compared with other species, and expression of some *LOXs* have been localized to the glandular trichomes where PCs are synthesized, suggesting that these may have acquired specialized roles in trichome biology and PC metabolism (Livingston et al., 2020; Borrego et al., 2023; Fayaz et al., 2023; Welling et al., 2023). Collectively, these data serve as evidence for the distinct yet incompletely understood role of the oxylipin pathway in PC production. In this review, we use a combination of phylogenetic and transcriptomic analyses to investigate the mechanisms by which oxylipins may influence PC production in *Cannabis* and propose a model highlighting these potential interactions.

## 2 *Cannabis:* a versatile and underutilized crop

### 2.1 Origin, classification and uses

*Cannabis* is a predominantly dioecious, herbaceous annual plant in the Cannabaceae family that originated in central Asia and now has a broad distribution (Kovalchuk et al., 2020). This genus comprises only one species but exhibits high polymorphism, with subspecies: *C. sativa* ssp*. sativa*, *C. sativa* ssp*. indica*, and *C. sativa* ssp*. rudelaris* characterized by varying chemical and morphological features (Small, 2015; Hazekamp et al., 2016). Although the plant is known for its intoxicating properties, it has been used over millennia for medicine, fiber, and oil extraction (Omare et al., 2021; Shakil et al., 2021). Current applications also extend to phytoremediation, functional foods, and as ornamental plants (Frassinetti et al., 2018; Wu et al., 2021; Hesami et al., 2022b). Classification of *Cannabis* has often been based on its end use, with recreational drug types typically referred to as ‘marijuana’ and fiber or seed types ‘industrial hemp’. Plants can also be classified into chemical phenotypes (chemotypes) based on their proportions of Δ^9^-tetrahydrocannabinol (Δ^9^-THC) or cannabidiol (CBD) PCs, or on the level of Δ^9^-THC, the principal intoxicant found in *Cannabis* (Jin et al., 2021; Hesami et al., 2022a; Salamone et al., 2022).

Despite the multiple end uses of *Cannabis*, research on this plant has been constrained over several decades due to its narcotic status, which has limited germplasm exchange and the application of modern genetic improvement strategies (Welling et al., 2016). Recent reforms to the cultivation and use of *Cannabis* by many legislators have enabled strong growth in licit *Cannabis* markets and these are projected to increase rapidly in the coming decades (de Brito Siqueira et al., 2023). Similarly, legislative reform has allowed for an increase in *Cannabis* research, with recent efforts focusing on establishing genomic resources for *Cannabis* and improving understanding on factors driving PC yield, such as flowering behavior as well as trichome initiation and hormonal regulation (Livingston et al., 2020; Hurgobin et al., 2021; Livingston et al., 2021; Apicella et al., 2022; Toth et al., 2022; Sands et al., 2023; Steel et al., 2023; Dowling et al., 2024; Huang et al., 2024).

### 2.2 PCs: important medicinal compounds

Extensive research has been conducted on the pharmacology of the two major PCs Δ^9^-THC and CBD, both of which have modulatory effects on the human endocannabinoid system (ECS). Δ^9^-THC is a partial agonist of type 1 G protein-coupled cannabinoid receptors (Tagen and Klumpers, 2022). These receptors have important roles in regulating memory, mood, sleep, appetite, inflammation, and pain sensation, and their activation has been shown to have analgesic, neuroprotective, and anti-nausea effects (Mechoulam and Parker, 2013). In contrast, CBD is also thought to modulate the ECS via a less understood mechanism involving other ECS targets and has been reported to counteract the intoxicating properties of Δ^9^-THC (Devinsky et al., 2014). Medicines containing Δ^9^-THC and CBD are currently used in the treatment of pediatric epilepsies (e.g., Dravet syndrome and Lennox-Gastaut syndrome) and to alleviate side-effects of chemotherapy, with many other PCs, such as the varinoid PCs cannabidivarin (CBDV; GWP42006) and tetrahydrocannabivarin (THCV; GWP42004), at various levels of drug development (Chanda et al., 2019; Urits et al., 2019; Britch et al., 2021). Other non-intoxicating and low abundant or minor PCs, including cannabichromene (CBC), cannabigerol (CBG) and their carboxylated analogues, cannabichromenic acid (CBCA) and cannabigerolic acid (CBGA), are also considered promising therapeutics (Borrelli et al., 2013; Anderson et al., 2021; Cabrera et al., 2021).

### 2.3 Glandular trichomes are multicellular structures that synthesize, secrete, and store enormous amounts of PCs

**Figure 2:**
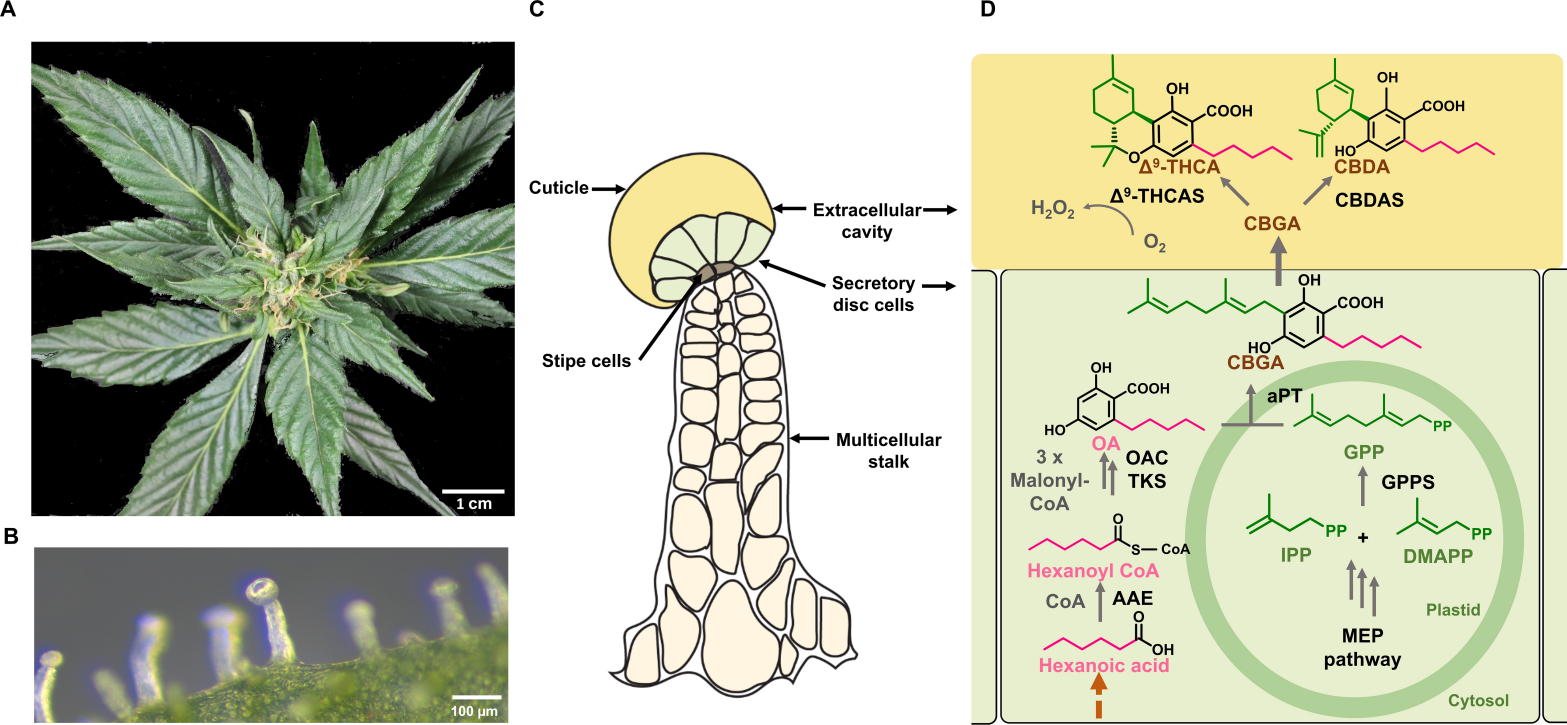
Spatial localization of the phytocannabinoid (PC) pathway in *Cannabis*. **(A)** Image of *Cannabis* female inflorescence. **(B)** Microscopic view of a capitate stalked glandular trichome on a perigonal bract. **(C)** Schematic representation of a capitate stalked glandular trichome, the major site of PC biosynthesis and storage. This consists of multicellular stalk (cream), stipe cells at the base of the trichome head (grey), secretory disc cells (light green) and large extracellular storage cavity (yellow). **(D)** The PC biosynthesis pathway in *Cannabis*. The methylerythritol phosphate (MEP) pathway takes place in non-photosynthetic chloroplasts of glandular trichome disc cells, while the polyketide pathway is localized in the cytosol. Geranyl pyrophosphate (GPP) is formed from the MEP pathway. The polyketide pathway requires an activated fatty acid starter unit (e.g., hexanoyl-CoA) and three molecules of malonyl-CoA and gives rise to olivetolic acid (OA). Prenylation with GPP by an aromatic prenyltransferase forms CBGA. This is exported to the extracellular cavity where cannabinoid synthases perform oxidative cyclization of the isoprenoid moiety of CBGA, forming the major acid PCs; CBDA and Δ^9^-THCA. In the final reaction, H_2_O_2_ is produced as a biproduct when CBGA is converted to PCs in the presence of O_2_. Solid dark grey arrows represent known steps in pathways while the brown dashed arrows represent steps of the pathway that have not been resolved. OA, olevetolic acid; IPP, isopentenyl diphosphate; DMAPP, dimethylallyl diphosphate; GPP, geranyl pyrophosphate; CBGA, cannabigerolic acid; CBDA, cannabidiolic acid; Δ^9^-THCA, Δ^9^-tetrahydrocannabinolic acid; AAE, acyl activating enzyme; TKS, tetraketide synthase; OA, olivetolic acid cyclase; GPPS, geranyl pyrophosphate synthase; aPT, aromatic prenyltransferase; CBGAS, cannabigerolic acid synthase, CBDAS, cannabidiolic acid synthase; D_9_-THCAS, D9-tetrahydrocannabinolic acid synthase.

*Cannabis* produces glandular as well as non-glandular trichomes on the surface of leaves and other parts of the plant body (Livingston et al., 2020; Xie et al., 2023). However, it is the capitate stalked glandular trichomes on modified leaves (perigonal bracts) surrounding female flowers that are the primary site for PC biosynthesis (**Figure 2A**). These trichomes are multicellular organs, consisting of a stalk, a disc of secretory cells, and a large globular head containing an extracellular cavity formed by cell wall delamination that stores the resinous compounds (Hammond and Mahlberg, 1973; Livingston et al., 2020; **Figures 2B, C**). These specific trichome morphotypes can produce substantial amounts of PCs, with these compounds contributing up to 40% of their dry weight (w/w) (Livingston et al., 2020). Capitate stalked glandular trichome initiation and development are coordinated with the onset of flowering and likely influenced by several phytohormones, including gibberellins, cytokinins, and JAs, as have been established in numerous plant species (Pattanaik et al., 2014; Li et al., 2021; Huang et al., 2024).

The biosynthesis of PCs traverses across several organelles within the secretory disc cells of glandular trichomes (**Figure 2D**). The precursors of PCs, olivetolic acid (OA), and geranyl pyrophosphate (GPP) are synthesized by the polyketide pathway in the cytosol and the methylerythritol 4-phosphate (MEP) pathway in plastids, while the final step occurs in the extracellular cavity (Livingston et al., 2022; **Figures 2C, D**). OA is formed by a type III polyketide synthase (tetraketide synthase; TKS), which produces a tetraketide intermediate, while the accessory protein, OA cyclase (OAC) catalyzes a C2-C7 aldol condensation (Gagne et al., 2012). Together, these enzymes form OA using three molecules of malonyl-CoA and an activated fatty acid, hexanoyl-CoA (Stout et al., 2012). Prenylation of OA with GPP produce the first PC, CBGA, a step catalyzed by an aromatic prenyltransferase, CBGA synthase (CBGAS) (Luo et al., 2019; Sands et al., 2023). CBGA is then transported to the extracellular cavity and converted to Δ^9^- THCA and CBDA by Δ^9^- THCA and CBDA synthases, respectively (Taura et al., 1996; Sirikantaramas et al., 2005). This compartmentalization is thought to be attributed to the cytotoxicity of PCs and their bi-product H_2_O_2_ which is formed at a molar ratio of 1:1 during the oxidative cyclisation of the isoprenoid residue of CBGA (Sirikantaramas et al., 2004, 2005; **Figure 2D**). The acidic PCs are then non-enzymatically decarboxylated over time or when subjected to heat, forming the bioactive neutral PCs, such as Δ^9^-THC and CBD (Sirikantaramas and Taura, 2017).

## 3 The origin of PC fatty acid precursors is unresolved

A key unanswered question in PC biosynthesis is the origin of the fatty acid hexanoic acid, which, once activated, serves as the C6 carbon starter unit for OA (Welling et al., 2023; **Figure 2D**). Several metabolic pathways could synthesize hexanoic acid within the secretory disc cells of capitate stalked trichomes. In the trichome glands of *Petunia*, C6-C8 straight-chain acyl acids are formed from branched-chain amino acid catabolism and α-ketoacid elongation (Kroumova et al., 1994). This pathway requires the activity of four proteins, yet transcripts for these genes have not been reported in *Cannabis* trichomes (Kroumova et al., 1994; Marks et al., 2009). A second pathway involves *de novo* fatty acid synthesis. This is supported by trichome-specific expressed sequence tags for a *3-oxoacyl- [acyl-carrier-protein (ACP)] reductase* (*fabG*), as well as the association of this gene with changes in PC composition in a genome-wide association study (Marks et al., 2009; Welling et al., 2020). While C6-specific thioesterases required to terminate fatty acid elongation have not been recovered from trichome cells, this enzyme could reduce the β-keto group following the first two condensation reactions of acetyl CoA, forming hexanoic acid, and, instead of leaving the condensing enzyme, participate in additional reactions with malonyl CoA (Horper and Marner, 1996; Stout et al., 2012). Contrary to this hypothesis is the functional validation of a cytosolic-localized hexanoic acid-specific AAE (hexanoyl-CoA synthetase) which is highly expressed in *Cannabis* trichome secretory disc cells (Stout et al., 2012).

**Figure 3:**
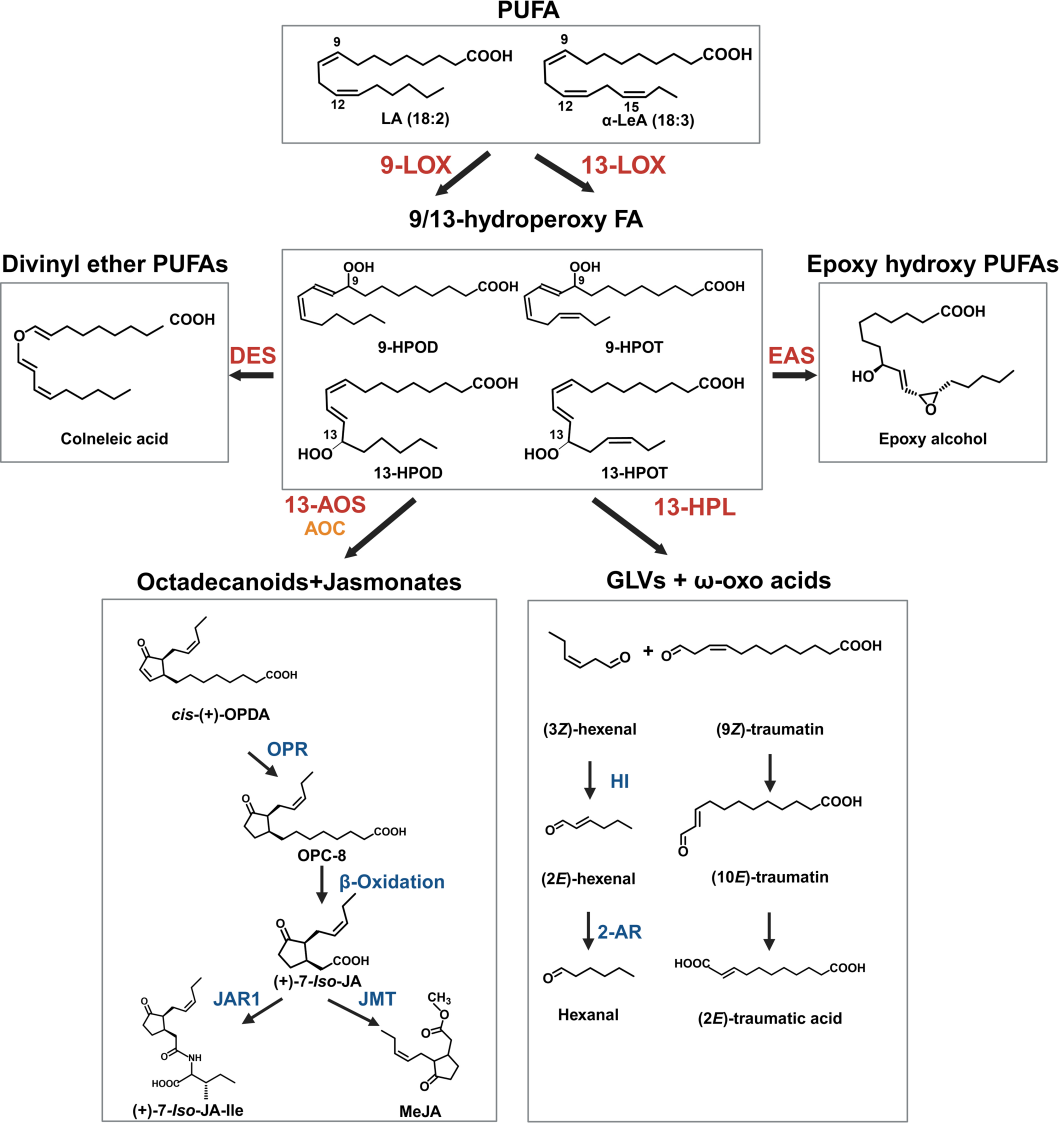
Key oxylipins in the oxylipin pathway. The oxylipin pathway starts with the hydroperoxidation of polyunsaturated fatty acids (PUFA) by lipoxygenase (LOX) enzymes. The position of oxygenation is either at the 9^th^ or 13^th^ carbon from the carbonyl end of the fatty acid. The resulting polyunsaturated hydroperoxides include 9-/13-hydroperoxy linoleic acid (9/13-HPOD) and 9-/13-hydroperoxy linolenic acid (9/13-HPOT). This reaction represents a key branchpoint for oxylipin biosynthesis and provides substrate for several enzymatic pathways, including allene oxide synthase (AOS), hydroperoxide lyase (HPL), divinyl ether synthase (DES), epoxy alcohol synthase (EAS) and peroxygenase (POX) pathways. The AOS and allene oxide cyclase (AOC) cascade leads to the formation of jasmonates (JAs), while the HPL branch leads to the production of green leaf volatiles (GLVs) and 12-oxo acids/traumatins. The other three pathways produce several groups of molecules that act in plant defense and signaling, including epoxy alcohols and divinyl ethers (e.g., colneleic acid). Abbreviations for compounds: α-LeA, α-linolenic acid; LA, Linoleic acid, 9-HPOD, (9*S*)-hydroperoxy-(10*E*,12*Z*)-octadecadienoic acid; 9-HPOT, (9*S*)-hydroperoxy-(10*E*,12*Z*,15*Z*)-octadecatrienoic acid; 13-HPOD, (13*S*)-hydroperoxy-(9*Z*,11*E*)-octadecadienoic acid; 13-HPOT, (13*S*)-hydroperoxy-(9Z,11*E*,15*Z*)- octadecatrienoic acid; epoxy alcohol, 13(*S*)-epoxy-9(*S*)-hydroxy-10(*E*)-octadecenoic acid; *cis*-(+)-OPDA, *cis*-(+)-12-oxo-phytodienoic acid; OPC-8, 3-oxo-2-(2-pentenyl)-cyclopentane-1-octanoic acid; (+)-7-*iso*-JA, jasmonic acid; (+)-7-*iso*-JA-Ile, jasmonic acid isoleucine conjugate;. (9*Z*)-traumatin, 12-oxo-(9*Z*)-dodecenoic acid; (10*E*)-traumatin, 12-oxo-(10*E*)-dodecenoic acid, (2*E*)-traumatic acid; (2*E*)-dodecanedioic acid; JAR1, jasmonoyl amino acid conjugate synthase; OPR, 12-oxo-phytodienoic acid reductase; AOC, allene oxide cyclase, HI, hexenal isomerase. 2-AR, 2-alkyl reductase.

A third hypothetical pathway could involve an oxylipin-based origin (Stout et al., 2012; Welling et al., 2023). This would involve the degradation of polyunsaturated fatty acids (PUFAs), such as linoleic or linolenic acid, and the activity of LOX to form hydroperoxy PUFAs that can be cleaved into C6 aldehydes and progenitors of hexanoic acid (Nakashima et al., 2013; Welling et al., 2023; **Figure 3**). Genes encoding desaturases, plastid-localized LOXs, and HPL that participate in these reactions are highly expressed in *Cannabis* trichomes (Stout et al., 2012; Balcke et al., 2017), while the secondary cleavage product of LOX/HPL, α-oxo acid 12-oxo-(10*E*)-dodecenoic acid ((10*E*)-traumatin) (**Figure 3**), has recently been correlated with PC content in *Cannabis* inflorescences following treatment with the oxylipin phytohormone MeJA (Welling et al., 2023; **Table 1**). C18 PUFAs and their oxylipin derivatives are also highly abundant in type VI stalked glandular trichomes of *Solanum* spp. and genes encoding acyl-hydrolyzing GDSL-type lipases that can release free C18 fatty acid substrates from galactolipids have been identified in *Cannabis* quantitative trait loci (QTL) for PC content (Cropano et al., 2022). The following sections provide a detailed discussion of oxylipins in *Cannabis* and highlight key areas of research that could be used to understand the interaction between oxylipins and PC production.

## 4 Oxylipin pathway and metabolism

### 4.1 Key metabolic branch points determine the fate of oxylipin molecules and their biological functions

In plants, oxylipins are generated through the oxidation of PUFAs. PUFA oxidation can occur enzymatically by dioxygenases or nonenzymatic by the activity of free radicals (e.g., singlet oxygen (^1^O_2_)) (Brash, 1999; Zoeller et al., 2012; **Figure 3**). The resulting hydroperoxides are highly reactive and act as key substrates in oxylipin metabolism, with the fate of oxylipin molecules decided by several enzymatic branch points including (1) the previously described HPL branch that forms C6 aldehydes, alcohols and acyl esters (collectively known as GLVs) concomitant with 12-oxo-(9*Z*)-dodecenoic acids ((9*Z*)-traumatin), (2) the JAs-forming AOS pathway, (3) the divinyl ether synthase (DES) pathway that forms divinyl ethers, and (4) the epoxy alcohol synthase/peroxygenase pathways that produce epoxy hydroxy PUFAs (Wasternack and Hause, 2013; Christensen et al., 2015, 2016; **Figure 3**). Oxylipins within each branch can have distinct roles (Schlotzhauer et al., 1996; **Figure 3**). For example, divinyl ethers and epoxy alcohols act as defense compounds in roots and leaves (Blée, 1998; Hamberg, 1999; Weber et al., 1999; Hamberg, 2004; Sanadhya et al., 2021; Gorina et al., 2022; Toporkova et al., 2024; **Figure 1**). The HPL and AOS branches are among the most extensively researched, with AOS-derived JAs having broad roles as signaling molecules, in reproduction, plant growth, and development (Scala et al., 2013; Ghasemi Pirbalouti et al., 2014; Ameye et al., 2018; Raza et al., 2021; **Figures 1**, **3**).

### 4.2 LOX serves as the critical juncture dictating oxylipin fate

LOXs, such as linoleate 13*S*-lipoxygenase (EC 1.13.11.12), serve as primary enzymes that mediate the fate of lipid precursors (**Figure 3**). These are monomeric non-heme iron (Fe)-containing dioxygenases widely distributed in plants that convert PUFAs into hydroperoxides in a two-step reaction, involving the reduction of Fe^3+^ to Fe^2+^ by proton-coupled electron transfer, followed by oxygen insertion (Egmond et al., 1973; Goldsmith et al., 2002). LOXs are categorized into two main functional classes based on the carbon position they catalyze. For the 9-LOX and 13-LOX this is at the 9^th^ and 13^th^ position from the carbonyl carbon in the fatty acid, respectively, and can also be distinguished as type I and type II based on subcellular localization (**Figure 3**). In addition, different LOX paralogs with the same regiospecificity can control different branches of the oxylipin pathway but are spatially separated. For example, in *Zea mays*, the JA-producing 13-LOX ZmLOX8 is localized to the chloroplast, while a GLV-producing 13-LOX ZmLOX10 is localized to non-photosynthetic plastids (Christensen et al., 2013). While often associated with defense process, impaired *LOX* function has resulted in changes in plant reproduction, fruit ripening and root development (Kolomiets et al., 2001; Fortes et al., 2004; Liu and Han, 2010; Caldelari et al., 2011; Vogt et al., 2013; Liu et al., 2020).

## 5 Phylogenetic insights and structural features of *Cannabis* LOX proteins

### 5.1 Phylogenetic analysis of the *Cannabis* LOX protein family reveals distinct clades that may have specific metabolic functions

**Figure 4:**
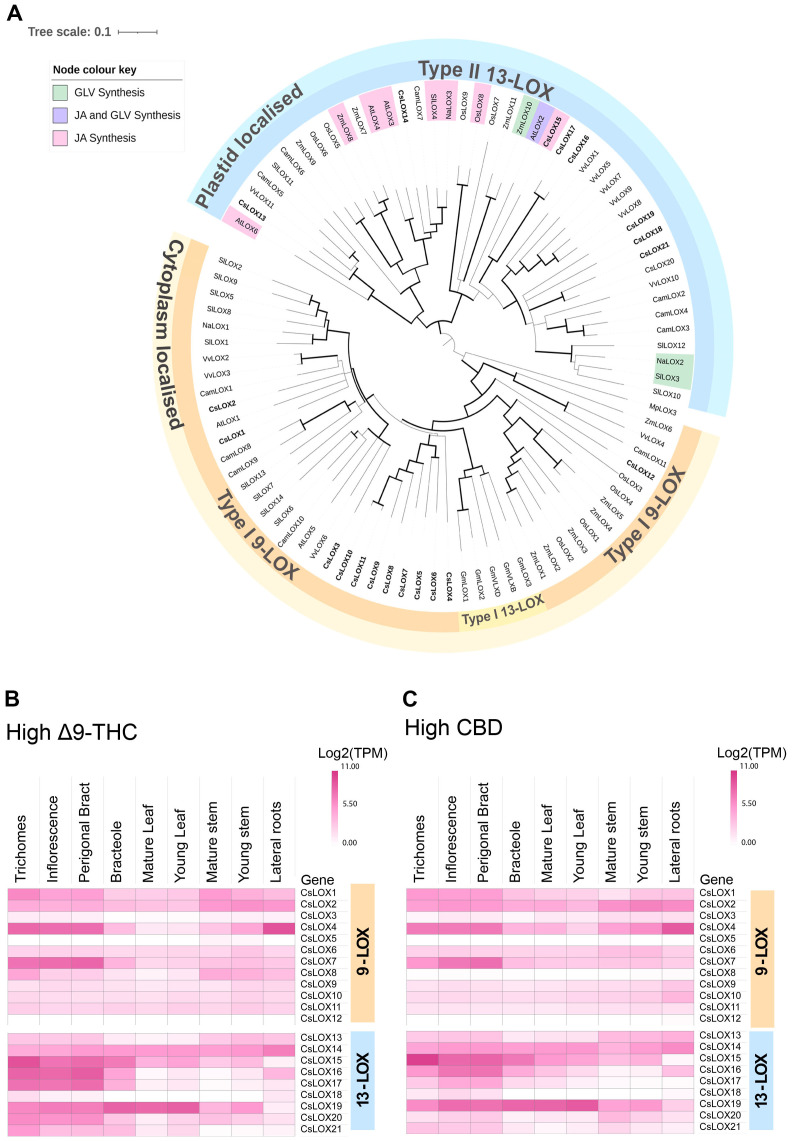
Phylogenetic relationship of LOX proteins and tissue series gene expression in *Cannabis*. **(A)** Phylogenetic relationship of lipoxygenase (LOX) proteins in *Cannabis* to several plant species, including *Arabidopsis thaliana* (AtLOX), *Camelia sinensis* (CamLOX), *Nicotiana attenuata* (NaLOX), *Oryza sativa* (OsLOX) *Solanum lycopersicum* (SlLOX), *Vitis vinifera* (VvLOX) and *Zea mays* (ZmLOX). Branches with Bootstrap values above 80 shown by greater line thickness (black). *Cannabis* LOX proteins are shown in bold. Functionally validated genes for corresponding LOXs are shaded as indicated by the key. Absolute expression (Log2 (mean TPM) of *LOX* genes in different tissue types of **(B)** high Δ^9^-THC and **(C)** high CBD chemotypes. RNA-Seq data reported by Jost et al. (2025). Protein sequences of *Arabidopsis thaliana* (At), *Cannabis sativa* (Cs), *Camelia sinensis* (Cam), *Nicotiana attenuata* (Na), *Glycine max* wild type (Gm) *Oryza sativa* (Os), *Solanum lycopersicum* (Sl) *Zea mays* (Zm) *and Vitis vinifera* (Vv) were used for phylogenetic analysis and PLAT/LH2 and lipoxygenase (LOX) domains identified using InterPro^1^ (v.98.0). Non-*Cannabis* sequences without both domains were removed from the analysis. Ninety-one higher plant LOX proteins were used for phylogenetic analysis. The *Marchantia polymorpha* (common liverwort) LOX3 protein served as an outgroup. The alignment was trimmed of spurious sequences and only residues of the Interpro lipoxygenase super family (IPR000907) domain was compared. The multiple sequence alignment was generated using clustal omega (ver. 1.2.2) in Geneious prime (ver. 2023.0.4), and the phylogenetic tree was generated using the neighbor-joining method (Sievers and Higgins, 2021) and visualized using Interactive Tree Of Life (iTOL; v. 6.8.1) (Letunic and Bork, 2007).

To explore the diversity of LOX gene family in *Cannabis*, a phylogenetic tree was constructed using protein sequences predicted from the *Cannabis* var. CBDRx genome [NCBI GenBank Accession: GCA_900626175.2] (**Supplementary Table S1**). Of the twenty-one *Cannabis* LOXs, all had the PLAT/LH2 domain (IPR001024) at the N terminus except CsLOX20 (**Table 2**, **Supplementary Table S1**). They also contained the C-terminal domain IPR013819 and were classified as LOX super family (IPR000907) members, which is consistent with authentic LOX proteins (Chen et al., 2015; Song et al., 2016). Twelve were clustered with the type-I cytosolic-localized 9-LOXs, while nine were clustered with the type-II plastidial-localized 13-LOX sub-class (**Figure 4A**). Within the 13-LOX sub-group, CsLOX13 and CsLOX14 form a clade with LOX orthologs associated with JA synthesis, while CsLOX15-CsLOX17 form a distinct clade that shows homology with AtLOX2, a dual functioning ortholog involved in both JA and GLV synthesis (Chen et al., 2004; Allmann et al., 2010; Chauvin et al., 2013; Christensen et al., 2013; Shen et al., 2014; Mochizuki et al., 2016; He et al., 2020; **Figure 4A**).

**Table 2:** LOC IDs of *Cannabis* 21 LOXs and different nomenclature used in other studies.

Nomenclature^1^	LOC ID	AA length	Major domains			Annotation	Nomenclature of genes used by different authors	
			Protein family IPR000907	PLAT/LH2 (IPR001024)	C Terminal (IPR013819)		Fayaz et al. (2023)	Welling et al. (2023)
CsLOX1	LOC115719608	860	21-860	18-161	163-860	probable linoleate 9S-lipoxygenase 5	CsLOX8	
CsLOX2	LOC115718693	955	116-955	113-256	258-955	probable linoleate 9S-lipoxygenase 5	CsLOX4	
CsLOX3	LOC115724062	873	32-873	29-172	174-873	probable linoleate 9S-lipoxygenase 5	CsLOX18	
CsLOX4	LOC115709296	859	18-859	15-160	162-869	probable linoleate 9S-lipoxygenase 5	CsLOX1	
CsLOX5	LOC115720291	848	34-848	5-149	151-848	probable linoleate 9S-lipoxygenase 5	CsLOX5	
CsLOX6	LOC115721132	859	42-859	18-160	162-859	probable linoleate 9S-lipoxygenase 5	CsLOX6	
CsLOX7	LOC115719336	871	29-871	21-163	165-871	probable linoleate 9S-lipoxygenase 5	CsLOX7	
CsLOX8	LOC115722276	869	51-869	19-161	163-869	probable linoleate 9S-lipoxygenase 5	CsLOX21	
CsLOX9	LOC115721268	875	52-875	25-167	169-875	probable linoleate 9S-lipoxygenase 5	CsLOX3	
CsLOX10	LOC115722275	868	45-868	18-160	162-868	probable linoleate 9S-lipoxygenase 5	CsLOX20	
CsLOX11	LOC115723988	868	45-868	18-160	162-868	probable linoleate 9S-lipoxygenase 5	CsLOX19	
CsLOX12	LOC115718785	707	1-707	1-51	54-707	linoleate 9S-lipoxygenase 1	CsLOX2	
CsLOX13	LOC115712696	935	129-935	54-236	238-935	lipoxygenase 6, chloroplastic	CsLOX17	LOX
CsLOX14	LOC115707105	931	125-931	97-236	238-931	linoleate 13S-lipoxygenase 3-1, chloroplastic	CsLOX1	
CsLOX15	LOC115719612	928	114-928	80-227	229-928	linoleate 13S-lipoxygenase 2-1, chloroplastic	CsLOX13	LOX-L
CsLOX16	LOC115719614	926	105-926	78-219	221-926	lipoxygenase 2, chloroplastic	CsLOX14	
CsLOX17	LOC115720530	926	105-926	78-219	221-926	lipoxygenase 2, chloroplastic	CsLOX15	
CsLOX18	LOC115719613	929	121-929	92-232	234-929	linoleate 13S-lipoxygenase 2-1, chloroplastic	CsLOX9	
CsLOX19	LOC115719615	922	105-922	92-228	230-922	linoleate 13S-lipoxygenase 2-1, chloroplastic	CsLOX12	
CsLOX20	LOC115719616	716	22-716	–	23-716	linoleate 13S-lipoxygenase 2-1, chloroplastic	CsLOX11	
CsLOX21	LOC115719617	906	83-906	75-212	214-906	linoleate 13S-lipoxygenase 2-1, chloroplastic	CsLOX10	

^1^ Borrego et al., 2023 LOX nomenclature.

### 5.2 Enhanced expression of *Cannabis LOX* in trichomes and floral tissues

We hypothesized that *LOX*s which have high expression in PC producing tissues, such as trichomes, perigonal bracts and inflorescences may have important roles in PC production. For example, some 9-LOX isoforms are exclusively expressed in roots where they participate in the synthesis of defense compounds (e.g., etherolenic acid, colnelenic acid) against root pathogens (Grechkin et al., 1997; Sanadhya et al., 2021). *CsLOX7*, *CsLOX15, CsLOX16* and *CsLOX17* consistently showed high levels of expression in the PC-producing tissues across two medicinal cannabis chemotypes, while *CsLOX15* was predominantly expressed in the trichomes (**Figures 4B, C**), indicating its potential involvement in trichome biochemistry. The recent heterologous expression of *CsLOX15* in *Nicotiana attenuata* and *N. benthamiana* plants indicate that this paralog encodes a functional enzyme, although activity was assessed using a single substrate, linoleic acid (Fayaz et al., 2023). Elucidating the molecular context of *CsLOX15* in its native environment will be critical to disentangle its precise role within *Cannabis* glandular trichomes and subsequent impact on PC production.

### 5.3 CsLOX15 structure - conserved residues in catalytic region

**Figure 5:**
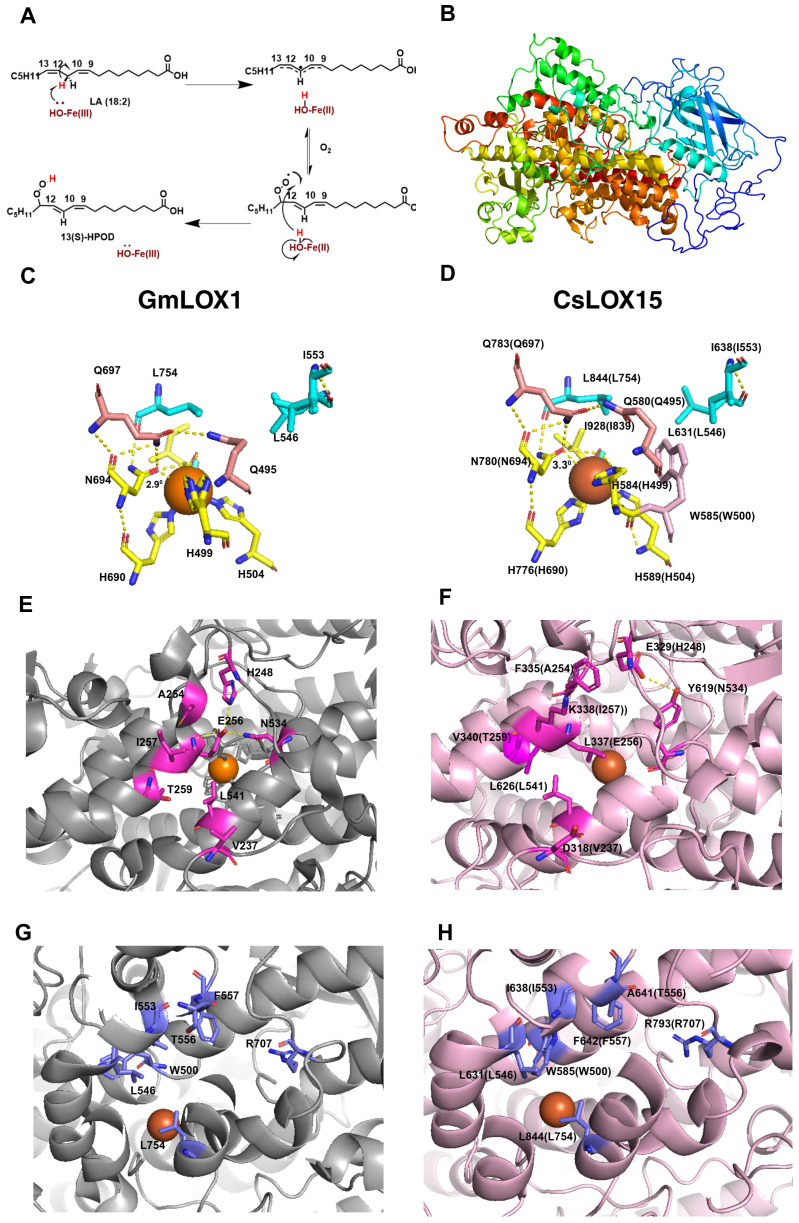
Structural features of GmLOX1 and CsLOX15. **(A)** Lipoxygenase (LOX) reaction mechanism, 13-LOX convert polyunsaturated fatty acids i.e. linoleic acid (LA) into (13*S*)-hydroperoxy-(9*Z*,11*E*)-octadecadienoic acid ((13*S*)-HPOD) by a two-step reaction involving the reduction of Fe^3+^ to Fe^2+^ by proton coupled electron transfer and then insertion of oxygen. **(B)** *In silico* 3D structure of CsLOX15 showing β- sheet rich N-terminal domain and α- helix rich C-terminal domain. **(C)** Coordination geometry of the catalytic site and Fe ligand of WT GmLOX1, showing important residues involved in iron coordination (H499, H504, H690, N694, I839, OH/H_2_O, Q495, Q697, N694) and substrate binding (I553, L546, L754). **(D)** *In silico* coordination geometry of CsLOX15, around the Fe ligand (superimposed to (5 A^0^)) showing iron coordination, substrate binding residues, and an additional side chain residue W585. **(E)** Amino acid residues in substrate entry of GmLOX1 facilitating H binding network. **(F)** Probable amino acid residues in CsLOX15 substrate entry. **(G)** Key amino acid residues within GmLOX1 substrate cavity. **(H)** Key amino acid residues within CsLOX15 substrate cavity. *Orange* sp*here*, LOX Fe ligand; *blue rod*, water molecule; *yellow dashed lines*, polar contacts. The secondary structure was predicted using Phyre2 (V 2.0) (normal mode) with 100% confidence (Kelley et al., 2015) and the validation of the model was carried out using PROCHECK program in SAVES v6.1^2^ (**Supplementary Figure 2**). The 3D-structure was modelled using PYMOL (Ver. 3.3) by superimposing with GmLOX1 (PDB entry 1F8N).

To further understand the structure and function of *CsLOX15* and other *Cannabis* LOXs, we compared the predicted structures of these *Cannabis* proteins with the crystal structure from the *Glycine max* (soybean) GmLOX1. As expected, many of the functional residues were conserved across the CsLOXs, with minor exceptions (Meyer et al., 2008; Offenbacher et al., 2017; Ruddat et al., 2004; Li et al., 2018) (**Supplementary Table S2**, **Supplementary Figure S1**). The predicted 3D structure of CsLOX15 shows the β-sheet rich N-terminal domain as well as the α-helix rich long carboxylic domain within the catalytic site, which facilitates electron transfer and the insertion of oxygen (**Figures 5A, B**, **Supplementary Figure S1**). The predicted 3D structure of CsLOX15, however, indicates that the side chain of W585 near the Fe ligand interacts with residue H584 (GmLOX1: H499) (**Figures 5C, D**). This may reduce space around Fe ligand by distorting the octahedral geometry, which could impact electron transfer (Steczko et al., 1992; Minor et al., 1996; Tomchick et al., 2001; Liavonchanka and Feussner, 2006; **Figure 5A**). We also observed that the N780 residue (GmLOX1: N694 involved in Fe binding) was positioned further away from the Fe ligand which could further weaken contact and increase flexibility (**Figures 5C, D**), Modification of N694H in GmLOX1 reduced flexibility due to stronger H bonding in the coordination sphere and resulted in reduced catalytic activity (Holman et al., 1998; Schenk et al., 2003; Segraves et al., 2006). The application of electron paramagnetic resonance (EPR) and magnetic circular dichroism (MCD) hydrogen deuterium exchange mass spectrometry will further aid in resolving the 3D structure and catalytic center of LOXs in non-model species such as *Cannabis* (Holman et al., 1998; Hu et al., 2019; Gaffney, 2020).

Analysis of the *in silico* predicted 3D structure of CsLOX15 also indicates changes in the substrate entry pocket which could potentially impact substrate specificity. Strong H bonds from residues H248-E256-N534 at the entrance of the substrate binding site of GmLOX1 have been partially lost in CsLOX15 (**Figures 5E, F**) (Skrzypczak-Jankun et al., 2001). Replacement of these with hydrophobic residues leucine and threonine as seen in CsLOX15 may alter the H bond network and expand the PUFA entry point (Youn et al., 2006). Replacement of V340 with the bulkier T259 residue (GmLOX1) also appears to have expanded the entry pocket (**Figures 5E, F**). Substrate orientation may also be affected in CsLOX15. For example, there was less distance between A641 (GmLOX1: T556) and R797 (GmLOX1: R707) within the substrate cavity which could potentially affect pocket volume, substrate entry and orientation (Hornung et al., 1999; Hughes et al., 2001; Hershelman et al., 2019; **Figures 5G, H**). Understanding the impact of these changes is complicated by the fact that there are no crystal structures of plant LOXs bound with linoleic acid or α-linolenic acid (Knapp and Klinman, 2003; Newie et al., 2016). Using *in-silico* tools such as Autodock to model the interaction between fatty acid and LOX proteins would provide deeper insights into substrate-enzyme interactions (Naimuzzaman et al., 2025).

Variations in the predicted tertiary structure of CsLOX15 compared to GmLOX1 suggest potential for differences in catalytic activity that might allow CsLOX15 to catalyze reactions utilizing numerous fatty acid substrates. As *Cannabis* glandular trichome disc cells are proposed to have non-photosynthetic chloroplasts, the role of CsLOX15 may facilitate the mobilization of storage lipids to provide carbon for producing defense compounds (Livingston et al., 2022). To meet the carbon demand, trichomes reliant on the supply of photosynthates from source organs, may be partially dependent on locally stored non-structural carbohydrates to maintain carbon supply (Huang et al., 2020). Hence, trichome-specific LOXs could be providing an alternate carbon source through hydrolysis of storage lipids.

## 6 The cytochrome P450 enzymatic step may represent an important branch point to study the effects of oxylipins on PC production

Both HPL and AOS are homologous cytochrome P450 enzymes that do not require molecular oxygen or the reducing power of NADPH reductase (Li et al., 2008). Structural analysis of the *A. thaliana* AOS protein shows that one point mutation in the catalytic site converts AOS to HPL (Li et al., 2008). While both enzymes compete for the same substrate (e.g. hydroperoxy fatty acids), competition can be controlled through temporal regulation, with changes in the time of expression observed in HPL- or AOS-specific LOXs in *N. attenuata*, or through spatial discrimination, as described previously in *Z. mays* and tomato (Froehlich et al., 2001; Allmann et al., 2010). Silencing *HPL* in soybean, rice, and potato impaired GLV production while increasing JAs synthesis, suggesting crosstalk between these pathways (Vancanneyt et al., 2001; Tong et al., 2012; Wang et al., 2020). As *Cannabis* is predicted to contain only a single copy of *HPL* (CYP74B) and *AOS* (CYP74A) (Borrego et al., 2023), silencing *CsHPL* or *CsAOS2* would be beneficial to determine compensatory mechanisms from loss of function of either gene and subsequent impact on PC production. To elucidate sequential steps up and downstream of these CYP74 enzymes, protein-protein interactions could also be explored by chemical cross-linking coupled with mass spectrometry (MS)-cleavable tag analysis (Piersimoni et al., 2021).

## 7 JAs and other oxylipins in PC production

### 7.1 The precise mechanism(s) by which JAs mediate PC production remains unresolved

There are several mechanisms by which JAs such as MeJA may increase PC content (**Table 1**). For example, through glandular trichome induction, increasing inflorescence compactness by reducing internode length, and/or the spacing of repeating phytomer units that host these specialized structures (Spitzer-Rimon et al., 2019; Welling et al., 2023; Huang et al., 2024). The complexity of *Cannabis* floral architecture makes measuring JA responses challenging. Consequently, there have only been limited attempts to quantify trichome density on floral leaves (e.g., perigonal bracts/calyxes), and there are mixed reports of the effectiveness of MeJA to induce trichomes on the foliar fan leaves (Welling et al., 2023; Hahm et al., 2024; Huang et al., 2024). Promisingly, a nearly two-fold increase in trichome density of calyxes was observed in 100 µM MeJA treated plants (Hahm et al., 2024). However, trichome imaging was limited to five calyxes per plant, which may not be representative, and density measurements were calculated based on a 6 x 4 mm leaf surface area (Hahm et al., 2024). The application of deep learning models and high spatial resolution imaging technologies could be used to accurately measure changes in floral architecture following exogenous JA exposure (Huang et al., 2024; Matsumoto et al., 2024).

JAs may also directly increase PC content by inducing PC gene expression and synthesis. To date, no significant changes in the expression of PC-related genes have been reported following a MeJA treatment (**Table 1**). However, spatiotemporal analysis of PC and precursor pathway genes within target tissues, such as the trichome secretory cells, following JA exposure are lacking (Garrido et al., 2022; Welling et al., 2023). JA related molecular responses are often associated with JA pathway genes (*LOX, AOS, AOC, JAZ* (*JASMONATE-ZIM-DOMAIN*)*, COI1* (*CORONATINE INSENSITIVE 1*) interacting with transcription factors like MYC2 and basic helix–loop–helix (bHLH)148, and a similar model is predicted for *Cannabis* (Schuurink and Tissier, 2020; Wang et al., 2020; Han et al., 2022; Song et al., 2022; Xie et al., 2023; Huang et al., 2024). The regulatory functions of JA signaling are typically initiated by the synthesis of JA-Ile, which promotes formation of the SCF^COI1^–JAZ co-receptor complex (Hickman et al., 2017). Degradation of JAZ repressor proteins by SCF^COI1^ results in the release of JAZ-mediated transcription factors, including MYC2, which activates the expression of JA-responsive genes by targeting their promoters (Hickman et al., 2017).

In *Cannabis*, the application of exogenous MeJA has increased the expression of JA-responsive genes like *JAZ* and *COI1* and the key regulator of these genes, *CsMYC4* (Huang et al., 2024). This suggests that *CsMYC4* may have a similar function to MYC orthologs in tomato and *L. angustifolia*, both of which influence glandular trichome size and density (Xu et al., 2018; Dong et al., 2022b; Huang et al., 2024). Members of the WRKY gene family, which have roles in glandular trichome formation in *Artemisia annua*, have also been shown to be responsive to MeJA in *Cannabis* (Xie et al., 2021; Rashid et al., 2023), indicating that a much larger network of transcription factors may be contributing to JA-mediated trichome formation. To fully understand these complexes, it will be necessary to comprehensively reconstruct the gene regulatory networks that form in response to JA exposure in *Cannabis*.

### 7.2 Mechanisms by which oxylipins may be interacting with PC production

**Figure 6:**
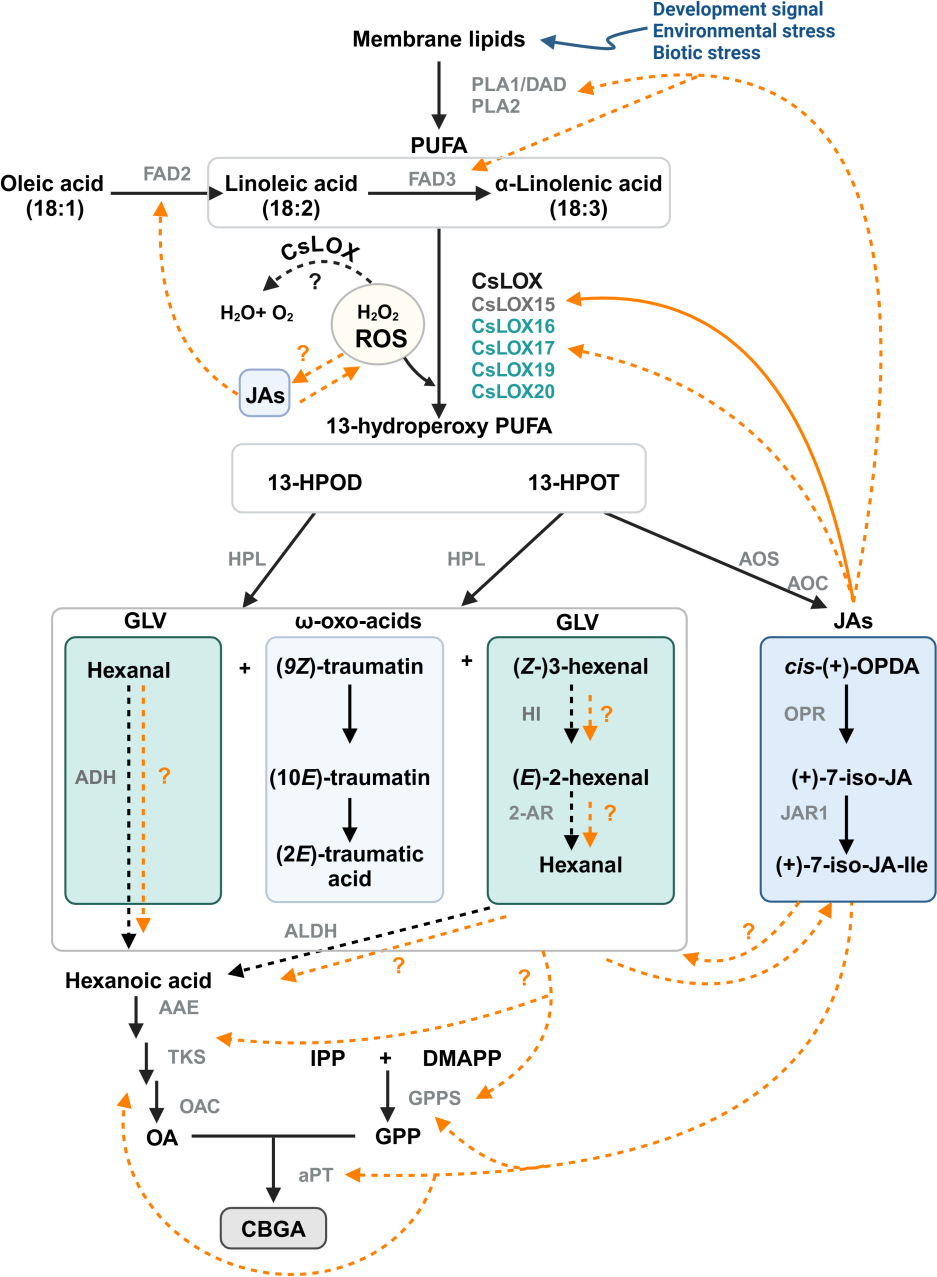
Proposed model for the interactions between oxylipin and phytocannabinoid metabolism in *Cannabis.* LOX-AOS and LOX-HPL branches of the oxylipin pathway may be interacting with phytocannabinoid biosynthesis. Potential mechanisms could include JA-mediated transcriptional regulation of phytocannabinoid biosynthesis genes or by providing a carbon source via the breakdown of PUFA. *Solid orange lines* represent known interactions; *orange dashed lines* indicate other possible interactions. Trichome-specific *Cannabis* LOX candidates for GLV and JA synthesis are shown (**Table 1**); *Black dashed lines* represent enzymatic reaction not yet fully explored in *Cannabis.* CsLOX15 (dark grey) has been functionally characterized, while other CsLOXs (turquoise) remain uncharacterized. Image created in BioRender.com PUFA, poly unsaturated fatty acids; 13-HPOD, (13*S*)-hydroperoxy-(9*Z*,11*E*)-octadecadienoic acid; 13-HPOT, (13*S*)-hydroperoxy-(9Z,11*E*,15*Z*)- octadecatrienoic acid; *cis*-(+)-OPDA, *cis*-(+)-12-oxo-phytodienoic acid; (+)-7-*iso*-JA, jasmonic acid; (+)-7-*iso*-JA-Ile, jasmonic acid isoleucine conjugate; (9*Z*)-traumatin, 12-oxo-(9*Z*)-dodecenoic acid; (10*E*)-traumatin, 12-oxo-(10*E*)-dodecenoic acid, (2*E*)-traumatic acid; (2*E*)-dodecanedioic acid; IPP, isopentenyl diphosphate; DMAPP, dimethylallyl diphosphate; OA, olivetolic acid; GPP, geranyl pyrophosphate; DAD, defective in anther dehiscence protein, a phospholipase (PLA1); FAD2/3, fatty acid desaturase 2/3; CsLOX, *Cannabis* lipoxygenase; HPL, hydroperoxide lyase; AOS, allene oxide synthase; AOC, allene oxide cyclase; OPR, 12-oxo-phytodienoic acid reductase; JAR1, jasmonoyl amino acid conjugate synthase; HI, hexenal isomerase; 2-AR, 2-alkynal reductase; ALDH, aldehyde dehydrogenase; AAE, acyl activating enzyme; TKS, tetraketide synthase; OAC, olivetolic acid cyclase; GPPS, geranyl pyrophosphate synthase; aPT, aromatic prenyltransferase.

Despite significant progress towards understanding the influence of oxylipin metabolism on PC production (**Table 1**), there are multiple avenues by which these lipid molecules may be driving metabolite production (**Figure 6**). The following interactions are proposed: (i) JAs could induce PC production through transcription regulatory mechanism, either indirectly through JA-induced responses, or directly by inducing the biosynthesis pathways. Similarly, HPL derived oxo acids and GLVs could interact with JAs and induce JA mediated responses or be directly involved as signal molecules inducing PCs, (ii) oxylipin-induced cleavage of fatty acids may form aldehydes that act as a direct carbon source for polyketide and PC synthesis, and (iii) oxylipins could be involved in the detoxification of reactive oxygen species (ROS) in the microenvironment of glandular trichomes (Balcke et al., 2017), as LOXs consume H_2_O_2_, the biproduct of PC synthesis (Taura et al., 1996; Sirikantaramas et al., 2005). ROS could also be generated in response to JAs and in return JAs can modulate ROS homeostasis (Denness et al., 2011; Corpas et al., 2015; Demiwal et al., 2024; Zhao et al., 2025). The interplay between ROS and JAs may lead to complex signaling pathways that could activate specialized metabolism in *Cannabis*. Additionally, JA signaling is closely aligned with the regulation of other phytohormones, including ethylene and salicylic acid, all of which can affect *LOX* expression and PC production (Yamamoto et al., 2020; Li et al., 2021; Jeyasri et al., 2023; Rodrigues Magalhães et al., 2023).

## 8 Conclusion

Despite being a multi-use crop with huge potential as a source for food, fiber and medicine, research on *Cannabis* has been constrained for more than 50 years. There exists a substantial knowledge gap on molecular drivers responsible for the diverse array of chemicals synthesized by *Cannabis* glandular trichomes. *Cannabis* has an extensive number of LOXs, and several are highly expressed in trichomes and are responsive to JAs (Fayaz et al., 2023). We identified the trichome-specific *CsLOX15*, which together with *CsLOX16* and C*sLOX17*, forms a distinct phylogenetic clade. The use of chemical inhibitors and gene silencing approaches, such as virus-induced gene-silencing (Schachtsiek et al., 2019; Alter et al., 2022), targeting these LOXs and other key branch points of oxylipin biosynthesis will be essential in understanding the contribution of oxylipins to PC production. Structure-function analysis of candidate LOXs by site-directed mutagenesis may also prove useful in understanding sequence variation among trichome-specific LOXs (Farmer et al., 1994; Li et al., 2020).

Determining the influence of oxylipin products among different trichomes types or tissues would also be interesting avenues for future research. However, due to high reactivity and volatile nature of these compounds, challenges exist in examining these in their native form. The use of spatial omics analyses of glandular trichomes and adjacent tissues may also help to resolve the contextual molecular components of these highly specialized cell types and aid in understanding of the molecular interactions between oxylipin and PC pathways. To trace the fate of oxylipin products, it will be necessary to use isotopic tracers to label PUFA precursors. Disentangling these interactions could provide important knowledge on the partitioning of carbon resources between primary and specialized metabolism and offer new opportunities for the biotechnological enhancement of *Cannabis*. These innovations could ultimately facilitate the development of elite chemical phenotypes for both industrial and medicinal applications.

## Footnotes

1
https://www.ebi.ac.uk/interpro/.

2
https://saves.mbi.ucla.edu.
