# Supplementary material for: Understanding the role of oxylipins in Cannabis to enhance cannabinoid production
Source: Front Plant Sci. 2025 Apr 24;16:1568548. doi: 10.3389/fpls.2025.1568548 (PMC12058684; doi:10.3389/fpls.2025.1568548)
Supplement: Supplementary file 1 [file DataSheet1.pdf]

Supplementary Material

Supplementary Table S1. UniProt and GenBank accession numbers of the proteins used for phylogenetic analysis

Species	Unique ID	Uniprot ID/NCBI accession No.	AA length	Protein family IPR000907	PLAT/LH2 domain IPR001024	C terminal domain IPR013819
Arabidopsis thaliana	AtLOX1	Q06327	859	20-859	19-162	164-859
	AtLOX2	P38418	896	103-896	73-200	202-896
	AtLOX3	Q9LNR3	919	112-919	84-223	225-919
	AtLOX4	Q9FNX8	926	118-926	90-229	231-926
	AtLOX5	Q9LUW0	886	55-886	33-181	183-886
	AtLOX6	Q9CAG3	917	110-917	80-217	219-917
Cannabis sativa	CsLOX1	XP_030504566.2	860	21-860	18-161	163-860
	CsLOX2	XP_030503376.2	955	116-955	113-256	258-955
	CsLOX3	XP_030509377.2	873	32-873	29-172	174-873
	CsLOX4	XP_030493230.2	859	18-859	15-160	162-859
	CsLOX5	XP_030505305.2	848	34-848	5-149	151-848
	CsLOX6	XP_030506244.2 isoform X1	859	42-859	18-160	162-859
	CsLOX7	XP_030504184.2 isoform X1	871	29-871	21-163	165-871
	CsLOX8	XP_030507302.2	869	51-869	19-161	163-869
	CsLOX9	XP_030506393.2	875	52-875	25-167	169-875
	CsLOX10	XP_030507301.2	868	45-868	18-160	162-868
	CsLOX11	XP_060960325.1	868	45-868	18-160	162-868
	CsLOX12	XP_030503471.2	707	1-707	1-51	54-707
	CsLOX13	XP_030496884.1 isoform X1	935	129-935	54-236	238-935
	CsLOX14	XP_030490813.2	931	125-931	97-236	238-931
	CsLOX15	XP_030504574.2 isoform X1	928	114-928	80-227	229-928
	CsLOX16	XP_030504577.2	926	105-926	78-219	221-926
	CsLOX17	XP_060967000.1	926	105-926	78-219	221-926

Supplementary Table S1. UniProt and GenBank accession numbers of the proteins used for phylogenetic analysis (cont.)

Species	Unique ID	Uniprot ID/NCBI accession No.	AA Length	Protein family IPR000907	PLAT/LH2 domain IPR001024	C terminal domain IPR013819
	CsLOX18	XP_030504576.2	929	129-929	92-232	234-929
	CsLOX19	XP_030504578.2	922	105-922	92-228	230-922
	CsLOX20	XP_060966996.1 isoform X1	716	22-716	-	23-716
	CsLOX21	XP_030504581.2	906	83-906	75-212	214-906
Camelia sinensis	CamLOX1	A8W7J7	861	18-861	17-161	163-861
	CamLOX2	B7TZ47	900	96-900	73-205	207-900
	CamLOX3	C4NZX3	901	98-901	73-206	208-901
	CamLOX4	A0A4S4EJA8	883	98-883	73-206	208-883
	CamLOX5	A0A2S1UFW5	914	79-217	79-217	219-914
	CamLOX6	A0A2S1UFW4	906	107-905	74-210	212-906
	CamLOX7	A0A2S1UFW3	912	98-912	75-214	216-912
	CamLOX8	A0A2S1UFW1	869	20-869	18-160	162-869
	CamLOX9	A0A2S1UFW6	906	58-906	56-198	200-906
	CamLOX10	A0A2S1UFX0	873	25-873	23-167	169-873
	CamLOX11	A0A2S1UFW8	876	54-876	30-156	186-876
Glycine max	GmLOX1	P08170	839	8-839	6-146	148-839
	GmLOX2	P09439	865	20-865	17-176	178-865
	GmLOX3	P09186	857	12-857	10-164	166-857
	GmLOXVB	Q43446	853	49-853	9-160	162-853
	GmLOXVD	P24095	846	53-864	10-172	174-864
Nicotiana attenuata	NaLOX1	A0A1J6IB64	861	20-861	18-164	163-861
	NaLOX2	Q6X5R6	900	90-900	70-204	206-900
	NaLOX3	A0A1J6HUT5	914	106-914	78-217	220-914

Supplementary Table S1. UniProt and GenBank accession numbers of the proteins used for phylogenetic analysis (cont.)

**Species**	**Unique ID**	**Uniprot ID/NCBI accession No.**	**AA Length**	**Protein family IPR000907**	**PLAT/LH2 domain IPR001024**	**C terminal domain IPR013819**
Oryza sativa	OsLOX1	Q76112	863	41-863	15-159	161-863
	OsLOX2	P29250	870	43-870	15-159	161-870
	OsLOX3	Q0IS17	868	45-868	21-152	154-868
	OsLOX4	Q53RB0	877	49-877	21-166	168-877
	OsLOX5	Q7XV13	899	96-899	66-205	207-899
	OsLOX6	Q8H016	918	108-918	98-219	221-918
	OsLOX7	Q2QNN5	922	101-902	76-203	205-922
	OsLOX8	P38419	924	103-924	88-223	225-924
	OsLOX9	Q84YK8	941	120-941	100-241	242-941
Zea mays	ZmLOX1	A0A3L6FBM0	873	51-873	21-167	169-873
	ZmLOX2	A0A1D6N521	871	51-871	21-167	169-871
	ZmLOX3	Q8W0V2	864	19-864	16-160	162-864
	ZmLOX4	A1XCH9	887	24-887	21-166	166-887
	ZmLOX5	A1XCI0	887	24-887	21-166	168-887
	ZmLOX6	A1XCI7	850	48-850	24-154	156-850
	ZmLOX7	B8XH56	916	107-916	79-218	220-916
	ZmLOX8	A1XCI3	941	110-941	82-221	223-941
	ZmLOX9	A1XCI4	922	116-922	86-226	228-922
	ZmLOX10	A1XCI5	905	103-905	68-209	211-905
	ZmLOX11	A0A8J8Y849	911	109-911	72-213	215-911
Solanum lycopersicon	SlLOX1	Solyc08g014000.2.1	860	18-860	17-160	162-860
	SlLOX2	Solyc01g099190.2.1	858	39-858	17-158	160-858
	SlLOX3	Solyc01g006540.2.1	896	93-896	67-198	200-896
	SlLOX4	Solyc03g122340.2.1	908	102-908	74-213	215-908
	SlLOX5	Solyc01g099160.2.1	862	18-862	17-160	162-862

Supplementary Table S1. UniProt and GenBank accession numbers of the proteins used for phylogenetic analysis (cont.)

Species	Unique ID	Uniprot ID/NCBI	AA Length	Protein family IPR000907	PLAT/LH2 domain IPR001024	C terminal domainIPR013819
	SlLOX6	Solyc09g075860.2.1	877	29-877	28-171	173-877
	SlLOX7	Solyc01g099200.2.1	841	14-841	10-146	148-841
	SlLOX8	Solyc08g029000.2.1	861	19-861	18-161	163-861
	SlLOX9	Solyc01g099180.2.1	854	14-854	11-152	154-854
	SlLOX10	Solyc12g011040.1.1	892	94-892	70-193	195-892
	SlLOX11	Solyc05g014790.2.1	911	82-911	77-215	217-911
	SlLOX12	Solyc01g006560.2.1	902	107-902	72-206	208-902
	SlLOX13	Solyc01g099210.2.1	863	28-863	26-167	169-863
	SlLOX14	Solyc09g075870.1.1	854	30-854	28-170	172-854
Vitis vinifera	VvLOX1	D5FUD8	901	76-901	70-205	207-901
	VvLOX2	F6HZ11	869	29-869	26-169	171-869
	VvLOX3	D5FUD9	859	18-859	16-159	161-859
	VvLOX4	A0A438GMK3	848	45-848	20-151	153-848
	VvLOX5	A0A438JV06	808	55-908	14-131	133-808
	VvLOX6	A0A438KLC1	892	56-892	31-173	175-892
	VvLOX7	F6GUA7	903	78-903	75-210	212-903
	VvLOX8	A0A438ELU2	904	76-904	71-208	210-904
	VvLOX9	A0A438ELV0	836	11-836	3-140	142-836
	VvLOX10	A0A438IAA9	876	105-859	78-210	203-866
	VvLOX11	A0A438I2F2	908	114-908	94-222	224-908
Marchantia polymorpha	MpLOX3	E5RS00	955	126-955	90-229	245-955

Supplementary Table S2: Key amino acid residues of GmLOX1 and comparison with Cannabis trichome specific LOXs

**GmLOX1**	**Function**	**9-LOX**	**13-LOX**	**Mode of determination**	**Reference**
H499	Fe ligand	Conserved	Conserved	X ray crystallography	Minor et al., 1996; Tomchick et al., 2001
H504	Fe ligand	Conserved	Conserved	X ray crystallography	Minor et al., 1996; Tomchick et al., 2001
H690	Fe ligand	Conserved	Conserved	X ray crystallography	Minor et al., 1996
N694	Fe ligand and second coordination sphere	Conserved	Conserved	X ray crystallography	Minor et al., 1996
I839	Fe ligand	Conserved	Conserved	X ray crystallography	Minor et al., 1996
Q495	Second coordination sphere to Fe	Conserved	Conserved	Mutant studies	Tomchick et al., 2001
Q697	Second coordination sphere to Fe	Conserved	Conserved	Mutant studies	Tomchick et al., 2001
L754	Second coordination and substrate binding	Conserved	Conserved	X ray crystallography	Hu et al., 2019
L553	Substrate binding pocket	Conserved	Conserved	Point mutations	Meyer et al., 2008
L546	Substrate binding pocket	Conserved	Conserved	Point mutations	Li et al., 2018; Hu et al., 2019
T556	Affecting substrate orientation	T (CsLOX1, CsLOX2, CsLOX7) V (CsLOX4)	A (CsLOX15, CsLOX19), T (CsLOX16), I (CsLOX17) S (CsLOX20)	Point mutations	Hornung et al., 1999; Hershelman et al., 2019
F557	Affecting substrate orientation	V	F	Mutation studies	Hornung et al., 1999; Hershelman et al., 2019
W500	Substrate binding	Conserved	Conserved	Point mutations	Ruddat et al., 2004
R707	Substrate positioning, substrate binding	Conserved	Conserved	Point mutations	Ruddat et al., 2004

Supplementary Table S2: Key amino acid residues of GmLOX1 and comparison with Cannabis trichome specific LOXs (cont.)

**GmLOX1**	**Function**	**9-LOX**	**13 LOX**	**Mode of determination**	**Reference**
S749	Surface loop H tunnelling	S (except CsLOX1-A)	A (CsLOx17-L)	Point Mutations	Offenbacher et al., 2017
Y317	Surface loop H tunnelling	F (CsLOX1. CsLOX2, CsLOX4, CsLOX7)	T (CsLOX15, CsLOX19, CsLOX20)<br>A (CsLOX16, CsLOX17)	Point mutations	Offenbacher et al., 2017
A542	Governing the stereospecificity	Conserved	Conserved	X ray crystallography	Youn et al., 2006
H248	Substrate entry strong H bond side chains	Conserved	Conserved	X ray crystallography	Youn et al., 2006
N534	Substrate entry strong H bond side chains	D	T	X ray crystallography	Youn et al., 2006
E256	Strong H bonding -side chains	A (CsLOX1, CsLOX2, CsLOX7), V (CsLOX4)	L (CsLOX15), S (CslOX16, CsLOX17), a (CsLOX19, CsLOX20)	X ray crystallography	Youn et al., 2006
T259	Substrate entry portal	L (CsLOX1, CslOX2, CsLOX4, CsLOX7)	V (CsLOX15, CsLOX20), M (CsLOX16) I (CsLOX17), L (CsLOX19)	X ray crystallography	Minor et al., 1996
L541	Substrate entry portal	F (CsLOX1), V (CsLOX2), L (CsLOX7)	L	X ray crystallography	Minor et al., 1996
V237	Cavity entry less bulky groups	N (CsLOX1, CsLOX2, CsLOX4), D (CsLOX7)	D (CsLOX15), A (CsLOX16, CsLOX17), R (CsLOX19), S (CsLOX20)	X ray crystallography	Youn et al., 2006
A254	Cavity entry less bulky groups	F	F	X ray crystallography	Youn et al., 2006
I257	Cavity entry less bulky groups	Y in all, N (CsLOX4)	K (CsLOX15, CslOX19) R (CsLOX16, CsLOX17)	X ray crystallography	Youn et al., 2006

```
Consensus MKTTXXLXXXXXXXXXXXXXXXXXXXXXXXXXXXXXXXXXXXXXXXXXXX    50
GmLOX1    --------------------------------------------------
GmLOX2    --------------------------------------------------
GmLOX3    --------------------------------------------------
GmVLXD    --------------------------------------------------
GmVLXB    --------------------------------------------------
AtLOX1    --------------------------------------------------
CsLOX1    --------------------------------------------------
CsLOX2    -----MLHYRNLNTPSPLLKFGAGVNS-LTSLVFKNSHNSLGFYSENHDR    44
CsLOX3    --------------------------------------------------
CsLOX4    --------------------------------------------------
CsLOX5    --------------------------------------------------
CsLOX6    --------------------------------------------------
CsLOX7    --------------------------------------------------
CsLOX8    --------------------------------------------------
CsLOX9    --------------------------------------------------
CsLOX10   --------------------------------------------------
CsLOX11   --------------------------------------------------
CsLOX12   --------------------------------------------------
CsLOX13   -----MLSLK--PTTTPLNNNPNISGDRSFSSISTATAGKDGDL-RRKTR    42
CsLOX14   ----MALAKE--IMCGSIIESTSFSSSASYSKVFLNQNF----R-RNQTR    39

                                  β1 (14-21)
CsLOX15   -----MLKPPHQVVQNLKCEKT------LVILNKPFIHGYNGAI-IG-IN    37
CsLOX16   -----MLKPHVQVTSGQT----------SVVLNKPFIHGNYHFQ-SP-FS    33
CsLOX17   -----MLKPHVQVTSGQT----------SVVLNKPFIHGNYHFQ-SP-FS    33
CsLOX19   MKTTEMLVNPTHHSNSSQILYR------RPPLRKTFVHGLDGAT-SS-FR    42
CsLOX18   -----MLMPQVMNLQTRPIQTTSFSRSFNNNQKSLFLLENNNI--GS-LS    42
CsLOX20   --------------------------------------------------
CsLOX21   -----MLKPQFQKSQTLLN---------SFCNKPLFIHGGNNL--SS-FP    33

Consensus XXXXXXXXXXXXXXXXXXXXXXXXXXXXXXXXXXXXXXXXXXXXXXXXXX   100
GmLOX1    --------------------------------------------------
GmLOX2    --------------------------------------------------
GmLOX3    --------------------------------------------------
GmVLXD    --------------------------------------------------
GmVLXB    --------------------------------------------------
AtLOX1    --------------------------------------------------
CsLOX1    --------------------------------------------------
CsLOX2    VLKSSCLIDLTRSQPLVGGGHCDRREESSISSSGEKMSVRCNTGS-----    89
CsLOX3    ----------------------------------------MGSGS-----     5
CsLOX4    --------------------------------------------------
CsLOX5    --------------------------------------------------
CsLOX6    --------------------------------------------------
CsLOX7    --------------------------------------------------
CsLOX8    --------------------------------------------------
CsLOX9    --------------------------------------------M-----     1
CsLOX10   --------------------------------------------------
CsLOX11   --------------------------------------------------
CsLOX12   --------------------------------------------------
```

```
CsLOX13    VAVSG--------SKLRRRGS----VRAAISSGDNKTETVSSNS------    74
CsLOX14    FLVSPVLVPLEQRRTLHLRKVV-RGPVAAISEDLVRSKAIQPSS------    82
CsLOX15    SRLFPV------KPK-------------------TKRRVASSSSSS----    58
CsLOX16    LLVP-----SKHTNK-------------------NN------------VG    47
CsLOX17    LLVP-----SKHTNK-------------------NN------------VG    47
CsLOX19    ILSLPK------RHK-------------------NNRRITCPTSTIRAIS    67
CsLOX18    SISNPN--PLLLVSA-------------------GNRARVSSSSS-----    66
CsLOX20    --------------------------------------------------
CsLOX21    VCII-R--PISLKNN-------------------NNKIIMKQKN------    55

Consensus  XXXXXXXXXXXXXXXXXXXXXXXXXXXXXXXXXXXXXXXXXXXXXXXXXX   150
GmLOX1     -----------------------MFSAGHKIKGTVVLMPKNELEVNP---    24
GmLOX2     ------------MFSVPGVSGILNRGGGHKIKGTVVLMRKNVLDFNSVAD    38
GmLOX3     -------------------MLGGLLHRGHKIKGTVVLMRKNVLHVNSVT-    30
GmVLXD     --------------------MFGIFDKGQKIKGTVVLMPKNVLDFNAITS    30
GmVLXB     --------------------MFPFGHKGQKIKGTMVVMQKNVLDINSIT-    29
AtLOX1     -------MFG-ELRD--LLTGGGNETTTKKVKGTVVLMKKNVLDFNDFNA    40
CsLOX1     -----------MLKDIIKAISGDNEEEGKKIRGSVVLMKKNVLDFNDFNA    39
CsLOX2     ----HSHNRI-SIEKEDGSGKTTTTKGSGKIKGNVVLMKKNVLDFNDFNA   134
CsLOX3     ----SPGITH-KVMQTFCMKDQKEETNHKKIQGEVVLMKKNILDLHDKSA    50
CsLOX4     --------------MFGIVKPPSIFGGNKKIKGNVVLMKKNVLDFNDMRN    36
CsLOX5     ------------------------MESGKKIKGVVILTKKNVLPRGTNAT    26
CsLOX6     -----------MVLNKIMDTLMNNGHGGKKFIGTVVLTKNNVIDHSALDR    39
CsLOX7     -------MLL-GKIGDRVSGILNRGDDQKRVKGSVVLMKKNVLEFNPLSA    42
CsLOX8     -------MFI-GK--VVDALSGGNNDDKKMINGKVVLSKKNVLEFNPLAT    40
CsLOX9     ----LLGKGI-GVVDSFTGGLIGGNNKGKKIKGSVVLVKKNVLEFNPLAS    46
CsLOX10    -----------MNLAGKLVDTLTGGLNGKKIKGRVVLAKKNVLEFNPLAA    39
CsLOX11    -----------MFLAGKVVDTLSGGLHGKKIKGRVVLAKKNVLEFNPLAA    39
CsLOX12    --------------------------------------------------
CsLOX13    SVPAHQSEDNS-------NGSLKKKKPSKGIEVRAVMTIRKKMKEK----   113
CsLOX14    SSPVESPV----------------ESKPVSFKVRAVVTVRNKNKED----   112

                     β2 (64-69)                 β3 (82-92)
CsLOX15    -SPGTK----NIIKA----SLFSPMEKKNTARVSVSVAVQRVTPKFWRFE    99
CsLOX16    VSISHRGKGNNIIKASLLGRLVSSKDKTATERVTVNVAVQRETAPL----    93
CsLOX17    VSISHGGKGNNIIKASLLGRLVSSKDKTATERITVSVVVQRETAPL----    93
CsLOX19    SSPSITDNFQEE------EEKRESEIEEETIAVKAVVT---VQPTVTGFF   108
CsLOX18    -NHSKRGLFVAPIKAILIGNIINKLTSN----VRLVVT---VLPTLGGVL   108
CsLOX20    --------------------------------------------------
CsLOX21    -----LGLGFNI------RASLSKDDDIKWIRVRVIVT---VHPTVGGML    91

Consensus  XXXXXXXXXXXXXXXXXXXXXXXXXXXXXXXXXXXXXXXXLXSXXXXXXX   200
GmLOX1     --------------------DGSAVDNLNAFLGR-SVSLQLISATKAD--    51
GmLOX2     LTKGNV----GGLIGTGLNVVGSTLDNLTAFLGR-SVALQLISATKPL--    81
GmLOX3     ----SV----GGIIGQGLDLVGSTLDTLTAFLGR-PVSLQLISATKAD--    69
GmVLXD     IGKGGVIDTATGILGQGVSLVGGVIDTATSFLGR-NISMQLISATQTD--    77
GmVLXB     ----SV----DGIVGTGLDFLGSALDTV-TFLAS-SISIQLISATKAD--    67
AtLOX1     ----------------------SFLDRLHEFLGN-KITLRLVSSDVTD-S    66
CsLOX1     ----------------------SILDRFHELVGK-GVSIQLISSVHGD-P    65
CsLOX2     ----------------------SFLDRVHELFGQ-RVSLQLVSAVNGD-P   160
CsLOX3     ----------------------SLLDRIYELFGK-GVSLHLISSVNPH-P    76
```

```
CsLOX4     -----------------------GIIDPLSELLGQ-RVSLQLISSVQT-HT    62
CsLOX5     SD--------------------SVVEPAAADISGQ-EVSLQLVSAVHG---    53
CsLOX7     SV----------------NVGSSLLDRVGDFLGN-GISIQLVSGH-----    70
CsLOX8     SV----------------NAGSAILDRVVEFLGG-GVSLQLVSGH-----    68
CsLOX9     SV----------------NTGTAIFDRFAEFLGS-GVSLQLVSGH-----    74
CsLOX10    SV----------------NAGSAIFDRLGEFLGN-GVSLQLVSGH-----    67
CsLOX11    SV----------------NAGSAMFDRLGEFLGN-GVSLQLVSGH-----    67
CsLOX12    --------------------------------------------------
CsLOX13    LA----------------EKMEDQWEFFVNGIGQ-GIQIQLISEEVDP--   144
CsLOX14    FK----------------ETLVKHLDAITDKIGR-NVVLELISTQTDP--   143

                                                  β4 (119-126)
                                      α1 (110-114)
CsLOX15    LS----------------EKIQDGRD[illegible]LGLNSLSIELVSTQKDP--   131
CsLOX16    -I----------------EKPQSLRDKTNDFLGVANFSVELISAQKDP--   124
CsLOX17    -I----------------EKPQSLRDKTNDFLGVANFSVELISTQKDP--   124
CsLOX19    KS----------------LGLDRGLDDIQDLLGK-TFLLELVSADLDP--   139
CsLOX18    TN----------------VGIGKGIDDAQDFFGH-SLLIELASAELDP--   139
CsLOX20    --------------------------------------------------
CsLOX21    SN----------------LGLKRGLDDTQDLLGR-SILIQLLSSQLDP--   122

Consensus  XXXXXXXXXXXXXXXXXXXXXXXXXXXXXXXXXXXXXXXXXXXXGXXXAX   250
GmLOX1     -AHGKGKVGKDTFLEGINT--SLPTLGAGESAFNIHFEWD-GSMGIPGAF    97
GmLOX2     -ANGKGKVGKDTFLEGIIV--SLPTLGAGESAFNIQFEWD-ESMGIPGAF   127
GmLOX3     -ANGKGKLGKATFLEGIIT--SLPTLGAGQSAFKINFEWD-DGSGILGAF   115
GmVLXD     -GSGNGKVGKEVYLEKHLP--TLPTLGARQDAFSIFFEWD-ASFGIPGAF   123
GmVLXB     --GGKGKVGKATNLRGKI---TLPTIGAKEEAYDAQFDWD-SDFGIPGAF   111
AtLOX1     ENGSKGKLGKAAHLEDWIT--TITSLTAGESAFKVTFDYE-TDFGYPGAF   113
CsLOX1     ENGLQGKLGKAAYLEDWIT--TITPLTAGESAFKVTFDLEDNDIGVAGAF   113
CsLOX2     GNGMRGKLGKEAYLEDWIT--TISPLTAGDSAFDVNFDWD-EKIGVPGAF   207
CsLOX3     EHVDRGSHGNAAYLEKWIT--ANTTITAKETCFNVFFDWE-ESMGIPGAL   123
CsLOX4     GNELKGKLGKESYLENWTNILTLFPLLAGESAYSVHFELE-EGFGVPEAF   111
CsLOX5     -NGVKGNVGEPAYLEKLDSY-DISTLSPRDIAFKVAFDKN-ESIGVPGAL   100
CsLOX6     ANGLKGKVGKEACLENEVTS-SLPSLAPGDNAFNVTLDLD-VSVGVPGAL   110
CsLOX7     ---SAGKVGKEAHLENWLT--SLPALTPGDSLFRVTFEWD-ESIGVPEAL   114
CsLOX8     ---NANKVGKETKLEDWVT--SAPTIIPGDSVFKVSFEWD-ESIGVPEAV   112
CsLOX9     ---TSGKLSKEAHLENWIT--SLPALTPGDSVFKVTFDWD-ESIGVPEAI   118
CsLOX10    ---VAGKVGKETHLENWVT--SLPTLTPGDSVFKVTFDWD-ESIGVPEAI   111
CsLOX11    ---TAGKLGKETHLENWVT--SLPTLTPGDSVFKVTFDWD-ESIGVPEAI   111
CsLOX12    -------------------------------------------MGVPPAL     7
CsLOX13    -VTNSGK-SIQSSVRGWLPKPT---NHLHIVEFAANFIVP-KDFGCPGAV   188
CsLOX14    -RTNGPKKSKEAVLRDWSKKVN---VKAERVNYTAEFEVD-SNFGIPGAI   188

                                      β5 (156-163)       β6 (172-178)
CsLOX15    -VTGKER-TVK----GFPKRPDFNIFSSDDVKYEAKFDIP-KDFGEVGAI   174
CsLOX16    -ETGKQRKM-K----CFPESKK-GGKKDKVMNYEAKVDIP-KDFGDVGAV   166
CsLOX17    -ETGKQRKM-K----CFPESKK-GGKEDKVMNYEAKVDIP-KDFGDVGAV   166
CsLOX19    -KTGEEKETVE----GYAHKIGKK---EDGIKYEASFKVP-IKFGHIGAI   180
CsLOX18    -KSGSERKRIK----GYAHKTSLI-DILGKVTYEVDFDIP-KEFGELGAI   182
CsLOX20    --------------------------------------------------
CsLOX21    -KTGLEKKTIN----KYAHRSSLW-N-KEGIQYETDLDVL-ADFGTVGAV   164
```

```
Consensus  XXXNXXXXXXXXXXXXXXXXXXXXXXXXXXXXXXXXXXXXXXXXXXXXXX   300
GmLOX1     YIKNYM-QVEFFLKSLTLEAIS-NQGTIRFVCNSWVYNTKLYK---SVRI   142
GmLOX2     YIKNYM-QVEFYLKSLTLEDVP-NQGTIRFVCNSWVYNTKLYK---SVRI   172
GmLOX3     YIKNFM-QTEFFLVSLTLEDIP-NHGSIHFVCNSWIYNAKLFK---SDRI   160
GmVLXD     YIKNFM-TDEFFLVSVKLEDIP-NHGTIEFVCNSWVYNFRSYK---KNRI   168
GmVLXB     YIKNYM-QNEFYLKSLILEDIP-NHGTIHFICNSWVYNSKHYK---TDRI   156
AtLOX1     LIRNSH-FSEFLLKSLTLEDVP-GHGRVHYICNSWIYPAKHYT---TDRV   158
CsLOX1     SIRNNH-HSEFYLKTLTLDY-P-DQTRVHFVCNSWVYPAKRYQ---KDRI   157
CsLOX2     IIQNYH-HSEFYLKTLTLDNVP-GHGRIHFVCNSWVYPSDKYH---SPRI   252
CsLOX3     MIKNNH-HSQFFLKTITLKDVP-GHGLVHFVCNSWVYPAHRYK---YDRI   168
CsLOX4     VINNNH-TTEFFLKSLTLEDVP-DQGQVHFVCNSWVYPANHYN---YDRV   156
CsLOX5     IIKNSG-SDEFYVKTITLVDVP-GEGHVHFVCNSWVYPFTKYD---YDRV   145
CsLOX6     IVTNNHATEKFFLKTFTLKDVP-DEGDIHFVCNSWIYPATKYD---YDRV   156
CsLOX7     IIKNNH-LDEFFLKTITLEDVP-GEGIVRFICNSWVYPAGRYK---HTRV   159
CsLOX8     IFKNNH-VEELFLKTITLDDVP-GEGVVQFICYSWVYSANKYD---YDRV   157
CsLOX9     IFKNNH-LDEFFLKTITLDDVP-AQGVVRFICNSWVYTANKYN---YNRV   163
CsLOX10    IFKNNH-VDEFFLKTITLDDVP-GQGVVRFICNSWVYSARKYN---YDRV   156
CsLOX11    IFKNNH-VDEFFLKTITLDDVP-GQGVVRFICNSWVYSARKYK---YDRV   156
CsLOX12    KITNNN-KDEFLLKYITLKYIX-----VKFLCNSWVYPQERYT---YPRV    48
CsLOX13    LVTNLH-GKEFYLLEIVIR--GFDGGPIFFLANTWIHSRNDNP---ESRI   232
CsLOX14    TVTNKH-QKEFFLEAITVE--GLACGPVHFPCNSWVQSKKDHP---AKRI   232

           β6             β7 (184-192)        β8 (200-204)          β9 (223-225)
CsLOX15    IVENDF-EREIFLKNIILEDLPSEPSTLEFSCNSWVQSKHDVPTDQHKRV   223
CsLOX16    IIENDF-EREIYMKTISLHYHSSDDKFVNFSCESWVQSKRDVPRDQQLRL   215
CsLOX17    IIENDF-EREIYMKTISLHYHSSDDKFVNFSCESWVQSKRDVPRDQQLRL   215
CsLOX19    LVENEH-HKEMYVSDIVLD--GLSNGPLNIICGSWVHSKFNNP---EKRI   224
CsLOX18    LVENEH-HKEMFLKDIKIDGEGLLNGPVTINCESWIHSKSQNP---QKRV   228
CsLOX20    --------------------------MIFKICLAKPF----FL---NLLA    17
CsLOX21    LVENEH-HKEMYIKNIVLQ--GFPNGPVNVTCNSWVHSKFDNP---QKRI   208

Consensus  XXXXXXXXPXXXXXXXXXXXRXXXLXXXRXXXXXGXXXXXXRXYDXXXYN   350
GmLOX1     FFANHTYVPSETPA-PLVSYREEELKSLRG-NGTGERKEYDRIYDYDVYN   190
GmLOX2     FFANHTYVPSETPA-ALVGYREEELKNLRG-DGKGERKEHDRIYDYDVYN   220
GmLOX3     FFANQTYLPSETPA-PLVKYREEELHNLRG-DGTGERKEWERVYDYDVYN   208
GmVLXD     FFVNDTYLPSATPA-PLLKYRKEELEVLRG-DGTGKRKDFDRIYDYDVYN   216
GmVLXB     FFANNTYLPSETPA-PLVKYREEELKNVRG-DGTGERKEWDRIYDYDVYN   204
AtLOX1     FFSNKTYLPHETPA-TLLKYREEELVSLRG-TGEGELKEWDRVYDYAYYN   206
CsLOX1     FFTNNTYLPSETPS-PLLKYREEELLSLRG-NREGELQEWDRVYDYAYYN   205
CsLOX2     FFANKTYLPSETPL-SLRKYREEELANLRG-NGTGERQEWDRIYDYDYYN   300
CsLOX3     FFSNKTYLPCKTPP-LLHHYRQEELKNLRG-NGTGELKEWDRVYDYDYYN   216
CsLOX4     FFRNKTYLPHQTPA-PLIWYREQELVYLRG-NGTGERKDGDRIYDYDFYN   204
CsLOX5     FFTNKSYLPSETPE-PLRQYRGQELLNLRG-NGTGELQVWDRVYDYAYYN   193
CsLOX6     FFRNKSYLPSETPE-PLRELREQELVNLRG-NGKGERQEWDRVYDYAYYN   204
CsLOX7     FFRNKSYLPSATPE-PLLKYRKEELESLRG-NGKGERKEWDRVYDYDVYN   207
CsLOX8     FFRNKSYLPSATPA-PLLKYRKEELQNLRG-DGKGERKEWDRVYDYDVYN   205
CsLOX9     FFRNKSYIPSATPA-PLLKYRKEELENLRG-NGLGQRKEWDRVYDYDLYN   211
CsLOX10    FFRNKSYLPSATPA-PLLKYRREELQNLRG-DGKGERKEWDRVYDYDVYN   204
CsLOX11    FFRNKSYLPSATPA-PLLKYRKEELQNLRG-NGLGERKEWDRVYDYDVYN   204
CsLOX12    FFTNKPYLPDNSERHKLHRLREGELRHLRSENNIGELKEWDRVYDYATYN    98
CsLOX13    IFKNQAYLPSQTPA-GLKDLRREDLLSIRG-NGKGQRKLHDRIYDYDVYN   280
```

```
CsLOX14     FFSNKPYLPSETPA-GLKVLREKELKKILRG-NGKGVRKLSDRIYDFDVYN    280

            β9                α2 (239-250)
CsLOX15     FFSNKCYLPSQTPS-GIKELRKIALENLRG-DGKGERKKNERVYDYDVYN    271
CsLOX16     FFSDKSYLPSQTPS-GLKELRNEELKRLRG-NGKGERKEYERVYDYDVYN    263
CsLOX17     FFSDKSYLPSQTPS-GLKELRNEELKRLRG-NGKGERKEYERVYDYDVYN    263
CsLOX19     FFTSKSYLPSQTPS-GLRRLRDEELVKLRG-NGEGERKHIDRIYDYDVYN    272
CsLOX18     FFTNKSFLPSNTPS-GLKKLREEDLKSLRG-NGEGQRKKHERIYDYDVYN    276
CsLOX20     LSSTLSYLPCETPS-GVRKLREEDLKNVRG-NGEGERKRFERIYDYDVYN     65
CsLOX21     FFTNKSYLPSQTPS-GLRRLREEELETLRG-NGRGERKFFERIYDYDVYN    256

Consensus   DJXXXXXXXXXXRXXJGGXXXXPXPRRXRTGRXXXXXXXXXXXXXXXXXX    400
GmLOX1      DLGNPDKSEKLARPVLGGSSTFPYPRRGRTGRGPTVTDPNTEKQG-----    235
GmLOX2      DLGNPDHGENFARPILGGSSTHPYPRRGRTGRYPTRKDQNSEKPG-----    265
GmLOX3      DLGDPDKGENHARPVLGGNDTFPYPRRGRTGRKPTRKDPNSESRS-----    253
GmVLXD      DLGNPDGGD--PRPILGGSSIYPYPRRVRTGRERTRTDPNSEKPG-----    259
GmVLXB      DLGDPDKGEKYARPVLGGS-ALPYPRRGRTGRGKTRKDPNSEKPG-----    248
AtLOX1      DLGVPPKN---PRPVLGGTQEYPYPRRGRTGRKPTKEDPQTESRL-----    248
CsLOX1      DLANPDKGPEYARPVLGGSSEYPYPRRGRTGRPPTKTDPNTESRL-----    250
CsLOX2      DLGNPDKGAKYARPVLGGSPQYPYPRRGRTGRKRTDTDRNTETRL-----    345
CsLOX3      DLGKPNK-PKYARPVLGGSEEHPYPRRGRTGRKKYKKYPEIETRL-----    260
CsLOX4      DLGNPDKGEKHVRPILGGTSEYPYPRRGRTGRPPTKTDPNSESRD-----    249
CsLOX5      DLGNPDKSPKLARQTLGGSSNFPYPRRGRTGRPPTQTDPNCESRL-----    238
CsLOX6      DLGNPDKGQDYVRQILGGSSDFPYPRRGRTGRPPTETDANIESSM-----    249
CsLOX7      DLGDPDKGSKYVRQTLGGSSEFPYPRRGRTGRAPNRTDPKTESRLKRVNL    257
CsLOX8      DLGEPDKGEDFARKILGGNSEFPYPRRGRTGRPPTKTDPKIESRLKAVNL    255
CsLOX9      DLGEPDKGRNFSRKTLGGNSEFPYPRRGRTGRPTTKSDSRTESRLKQVNL    261
CsLOX10     DLGEPDKGSNFVRKILGGNSEFPYPRRGRTGRASTKTDSTSESRLKQVNI    254
CsLOX11     DLGEPDKGSNFVRKTLGGNSEFPYPRRGRTGRAPTKTDSTSESRLKQVNI    254
CsLOX12     DLGN-------NRPILGGK-DLPYPRRGRTGRPLTHDGHESRPDK-----    135
CsLOX13     DLGKPEN-KDLARPVIGGE-KRPYPRRCRTGRPPSKSDPLSETRI-----    323
CsLOX14     DLGNPDKSTELARPKLGGK-EIPYPRRCRTGRLPTDTDLQAESRI-----    324
CsLOX15     DLGQPDNNDDLKRPVLGGSKEFPYPRRCRTGRPPTETDPLSESRI-----    316
CsLOX16     DLGDPDKTSDLTRPVLGGS-QRPYPRRCRTGRPPAKTDLSSESRS-----    307
CsLOX17     DLGVPDKSSDLTRPVLGGP-QHPYPRRCRTGRPQTKTDLSSESRS-----    307
CsLOX19     DIGDPDRKFKLSRPVLGGE-ERPYPRRCRTGRPPCESDTLAEKRS-----    316
CsLOX18     DLGSPDLMSDLKRPVLGGK-EHPYPRRCRTGRHPSRSDPKTESRL-----    320
CsLOX20     DLGNPDKSHDLKRPVLGGK-ERPYPRRCRTGRPRTKTDPVSEQNS-----    109
CsLOX21     DLGDPDKSEDLKRPVLGGK-NRPFPRRCRTGRPPTQRDPLSEQPS-----    300

Consensus   XXXXXXXXXXXXYXPRDEXXXXXKXXXXXXXXXXXXXXXXXXXXXXXXXX    450
GmLOX1      ---------EVFYVPRDENLGHLKSKDALEIGTKSLSQIVQPAFES-AFD    275
GmLOX2      ----------EVYVPRDENFGHLKSSDFLAYGIKSLSQYVLPAFES-VFD    304
GmLOX3      ---------NDVYLPRDEAFGHLKSSDFLTYGLKSVSQNVLPLLQS-AFD    293
GmVLXD      ----------EVYVPRDENFGHLKSSDFLTYGIKSLSHDVIPLFKSAIFQ    299
GmVLXB      ---------DFVYLPRDEAFGHLKSSDFLAYGIKSVAQDVLPVLTD-AFD    288
AtLOX1      ----PITSSLDIYVPRDERFGHLKMSDFLAYALKAIAQFIQPALEA---V    291
CsLOX1      ----PILMSLNIYVPRDERFGHLKMSDFLAYALKSVAQVIKPELES---K    293
CsLOX2      ----NLALSLNIYAPRDERFGHLKLADFLAYALKSIGQFLKPEIED---L    388
CsLOX3      ----PL-LNLDIYVPRDERFGHVKFSDFLAYALKSLVQVLIPELKY---L    302
CsLOX4      ----IDLLNLNFYIPRDERFGHLKMSDFLVNTLKSVALIIKPAIES---L    292
```

```
CsLOX5     ----DIKKSLTIYVPRDEQFNHVKMSDFLAYGLLSIAQAIKPALES---Y   281
CsLOX6     ----SLITSLNVYVPRDERFGHVKMADFLGYGLKSLAQGIFPALES---Y   292
CsLOX7     MKPLDPLEALDIYVPRDERFGHLKMSDFLAYGLKSLSQSIKPALEH---Y   304
CsLOX8     TKPLDPVESLDIYVPRDERFGHLKMSDFLAYAIKALSHAVVPALKH---F   302
CsLOX9     MNPLDPVESLDIYVPRDERFGHLKMSDFLAYGIKSLTQAIIPALKH---Y   308
CsLOX10    MKPLDPIESLDIYVPRDERFGHLKMSDFLAYAIKSLSQAIVPALKH---F   301
CsLOX11    MKPLDPIESLDIYVPRDERFGHLKMSDFLAYAIKSLSQAIIPALKH---F   301
CsLOX12    --------NQVIYIPRDEQINHLKTEPPPSSD--TVAKIITTLSED---D   172
CsLOX13    ------EKPHPVYVPRDETFEEIKQNTFSAGRLKALLHNLIPSIAA---S   364
CsLOX14    ------EKPFPLYVPRDEQFEESKQASFSFGRLKAVLHNLIPSLKA---S   365

                                            α3 (340-352)
CsLOX15    ---------SDFYVPRDEEFAEVKQSNFSLKTVYSVIHAVIPILRQ---V   354
CsLOX16    --------DKAFYVPRDETFAEVKQSDFGSRRMQLALKTLLPFLEN---N   346
CsLOX17    --------KTAFYVPRDETFAEVKQNDFASRRIALGIKSLIPFLES---N   346
CsLOX19    ---------GRFYVPRDECFSEIKQLTFSAKTLYSVLHALSPSLAN---V   354
CsLOX18    ------SSDVLFYIPRDENFSEIKQMTFGAKTLFSVMHALVPSLEG---L   361
CsLOX20    ---------ASVYVPRDEAFSEIKQITFSANTVYSGLHALVPSLQT---A   147
CsLOX21    ---------RTIYVPRDETFSEIKQLTFQAKTIYSGLHALVPSLQT---V   338

Consensus  XXXXXXXXXXXXXXXXXXXXXXXXXXXXXXXXXXXXXXXXXXXXXXXXXX   500
GmLOX1     LKSTPIEFHSFQDVHDLYEGGIKLPRD----VI--STIIPLPVIKE----   315
GmLOX2     LNFTPNEFDSFQDVRDLHEGGIKLPTE----VI--STIMPLPVVKE----   344
GmLOX3     LNFTPREFDSFDEVHGLYSGGIKLPTD----II--SKISPLPVLKE----   333
GmVLXD     LRVTSSEFESFEDVRSLYEGGIKLPTD----IL--SQISPLPALKE----   339
GmVLXB     GNLLSLDFDNFAEVRKLYEGGVTLPTN----FL--SNITPIPIIKE----   328
AtLOX1     FDDTPKEFDSFEDVLKIYEEGIDLPNQALIDSI--VKNIPLEMLKE----   335
CsLOX1     FDNTPSEFDSFQDVLNMYDGGIELPKG-LVDDI--RENIPAEMLKE----   336
CsLOX2     FNSTPNEFDSIDDVFKLYEGGVDVPEG-LLKSV--RDNIPAEMLKE----   431
CsLOX3     TNKTFNEFDSFKQVLELYDDEAKKPKGHTLSKI--RECVPCELLRE----   346
CsLOX4     FDRTPAEFDSFKDVLSLYEGGFPLPLT-VFETI--SKNVPLETLKD----   335
CsLOX5     FDETPGEFDSFQDVLDLYKGGLKLPKE-VVDNI--KKDVPSEMLEE----   324
CsLOX6     FGETPGEFSSFEEVYDLYKGGLKLPTT-VVDNI--EKNVPAELLQE----   335
CsLOX7     FDQTRNEFDNFQEVYDLYEGGFKLPTA-VLDTI--RKTVPFNMLKE----   347
CsLOX8     FDETPNEFDKFQEVHELYEGGLELPTK-VFDKI--RKAVPDDMLKE----   345
CsLOX9     FDQTRNEFDNFQEVLDLYEGGLKLPKS-VLDNI--RKNVPFDLLKE----   351
CsLOX10    FDETRNEFDSFKEIEDLYEGGLKLPTS-VLSTI--RNNVPVDLFRE----   344
CsLOX11    FDQTRNEFDSFKEIDDLYEGGLKLPTS-VLNTI--RNNVPVDLFRE----   344
CsLOX12    TVLDDNYYRSFREVLALFQGT-----------------------------   193
CsLOX13    LSSSDISFNCFTDIDKLYNDGFFLKDEDQNEVS--------FTFPVMGKF   406
CsLOX14    FSAENQEFSGFADIDNLYSEGILLKLGVQEELL-----------KKLPLN   404

                         α4 (366-371)          α5 (382-388)
CsLOX15    LI--DENFPYFTAIDVLYDEGIKIPSNAEKSLI--------QTIKNVNAR   394
CsLOX16    MF--DEKFPNFTDIDKMFKEGYEITDKSISGDKSLKEMLLLHVGTKLHSS   394
CsLOX17    MF--DEKFPNFTDIDKMFKEGYEITDKSISGDKSIKEMLALHVGTKLYSS   394
CsLOX19    MADKDLGFPYLTAIDALFSKGVDLPTIGT-E----------GFLRKVMPR   393
CsLOX18    LV-DKEGFPYFTAIDTLFDEGIKIPPNHHNK----------SLLKSALPR   400
CsLOX20    LLNPSLGFPYFRAIDSLFDDGLKLPPTKIEQ----------GILRNVLPR   187
CsLOX21    LTDSSLGFPYFRAIDSLFDNGLKLPPSTN-Q----------GLLKNILPR   377
```

```
Consensus  XXXXXXXXXXXXXXXPXXXXXXXXXXWXXDXEFXRZXXAGXXPXXJXXX   550
GmLOX1     --LYRTDGQHILKFPQPHVVQ-VSQSAWMTDEEFAREMIAGVNPCVIRGL   362
GmLOX2     --LFRTDGEQVLKFPPPHVIQ-VSKSAWMTDEEFAREMVAGVNPCVIRGL   391
GmLOX3     --IFRTDGEQALKFPPPKVIQ-VSKSAWMTDEEFAREMLAGVNPNLIRCL   380
GmVLXD     --IFRTDGENVLQFPPPHVAK-VSKSGWMTDEEFAREVIAGVNPNVIRRL   386
GmVLXB     --LFRTDGEQFLKYPPPKVMQ-VDKSAWMTDEEFARETIAGLNPNVIKII   375
AtLOX1     --IFRTDGQKFLKFPVPQVIK-EDKTAWRTDEEFAREMLAGLNPVVIQLL   382
CsLOX1     --IFRTDGERLLKYPLPQVLK-ESRSAWRTDEEFAREMLAGVNPVSISLL   383
CsLOX2     --IFRTDGERFLKFPVPQVIK-ENKTAWDTDEEFAREMLAGINPVMIHRL   478
CsLOX3     --ILR-QEEGFRKLPMPDVIR-EDNSAWRTDEEFGREMLAGVNPVIIRRL   392
CsLOX4     --LFRADGTRFLKYPMPHVIK-EDKAAWMTDEEFGREMLAGVNPVVIRRL   382
CsLOX5     --IFHIEAGQFIKYPLPHVIK-EDTSAWRTDEEFGREMVAGVNPLVIRRL   371
CsLOX6     --MFKIDRGQFLKYPPPHVIK-ENKSGWRTDEEFGREMVAGVNPVVIRRL   382
CsLOX7     --LFRTDGEQFLRFPVPHVIK-VDKSAWRTDAEFGREMLAGVHPIAIRRL   394
CsLOX8     --LLRSDGEKFLEFPMPDVIK-ESKSAWRTDEEFGREMLAGVHPVLIASL   392
CsLOX9     --MFRTDGEQFLKFPLPHVIK-EDKSAWRTDEEFGREMVAGVHPVLIRRL   398
CsLOX10    --LLRTDGEQFLKFPMPAVIK-ESKSAWRTDEEFGREMVAGVHPILIRRL   391
CsLOX11    --ILRTDGEQFLKFPMPAVIK-ESKSAWRTDEEFGREMVAGVHPILIRRL   391
CsLOX12    ------------GLPVPGVIEAGDENAWRDDEEFGREMIAGVHPVHIRLL   231
CsLOX13    MKQVMSVQERLFKYDVPAVIR-RDRFSWLRDNEFARQCLAGVNPVSIELL   455
CsLOX14    LVTRIQQNKGILKYDTPKIIS-KDKFAWLRDDEFARQAIAGVNPVSIERM   453

                 α6 (401-405)                 α7 (425-432)  β10 (438-442)
CsLOX15    IYKTVSDADDFLQFQQPPTMD-KDKFFWFRDEEFCRQTIAGLNPCCIELV   443
CsLOX16    VLVAESLVDNVIKFKQPPTAD-MDKFFWFRDEEFARQTIAGLNPCCLQLV   443
CsLOX17    VLVAESLVDNVIKFKQPPTAD-MDKFFWFRDEEFARQTIAGLNPCCLQLV   443
CsLOX19    VVKTMDSGKDVLRFEPPETIN-RDKLFWFRDEEFARQTLAGLNPCSIRLI   442
CsLOX18    LVKAASDVDDVLQFVPPEPMD-RDKFFWLRDEEFGRQTLAGLNPHGIQLV   449
CsLOX20    LIKTVKDANDALQFEIPDTLD-RDKFFWFRDEEFGRQTLAGLNPCCIQLV   236
CsLOX21    LVKTVSDANEALQFEIPEPMD-RDKFFWFRDEEFGRQTLAGLNPYSIQLV   426

Consensus  XXXPXXSXLBXXXXGXXXXXJXXXXXXXXXXXXXXXXXXXAXXXXXXXXX   600
GmLOX1     EEFPPKSNLDPAIYGDQSSKITADSL--DLD--GY-TMDEALGSRRLFML   407
GmLOX2     QEFPPKSNLDPTIYGEQTSKITADAL--DLD--GY-TVDEALASRRLFML   436
GmLOX3     KEFPPRSKLDSQVYGDHTSQITKEHLEPNLE--GL-TVDEAIQNKRLFLL   427
GmVLXD     QEFPPKSTLDPTLYGDQTSTITKEQLEINMG--GV-TVEEALSTQRLFIL   433
GmVLXB     EEFPLSSKLDTQAYGDHTCIITKEHLEPNLG--GL-TVEQAIQNKKLFIL   422
AtLOX1     KEFPPKSKLDSESYGNQNSTITKSHIEHNLD--GL-TVEEALEKERLFIL   429
CsLOX1     QEFPPASKLDKNTYGDQTSTITEEHIKNNLD--GL-TVNEALEKNKLFIL   430
CsLOX2     QEFPPRSKLDPKVYGDHTSKITKNYIEHNLD--GL-SVDEAIRNNKVFIL   525
CsLOX3     EVFPPVSTLDATQYGKQDSTIIEEHIEAYMN--GL-TVSEAIEQNKLFIL   439
CsLOX4     QEFPPTSKLDPSIYGNQTSNITESHIINNLD--GL-SVNEALEQNKLYIL   429
CsLOX5     EEFPPTSKLNPQVYGNQTSKITEEHILNHLD--GL-VVHEALKSKRLFIL   418
CsLOX6     EEFPPKSTLDPKVYGDQTSKIIEDHIQSNLD--GL-GVFEALNNNKLFIL   429
CsLOX7     EEFPPASKLDHKVYGDQTSKITEDHIEKNLE--GL-SVYEALYTNKLFIL   441
CsLOX8     KEFPPTSKLDKKLYGDHTSKITKEHIQNYLE--GL-DVDTALNQNKLFIL   439
CsLOX9     EEFPPTSKLDPELYGDHTSTITEDHIKNSLE--GL-GVFEALFQNKLFIL   445
CsLOX10    NEFPPASKLDPELYGDQTSTITEEHIQNYLD--GL-DVSTALSQNRLYIL   438
CsLOX11    NEFPPASKLDPGLYGDQTSTITEEHIQNYLD--GL-DVSTALNQNRLYIL   438
CsLOX12    KKFPPKSKLDRRKYGDQDSRISEDDIIKMLE--DFSSVEKAIEQKRLFIL   279
CsLOX13    KEFPILSKLDPAKFGAPESAITKELLEQEL---GGMSVEKAIEDKKLFIL   502
```

```
CsLOX14    TVFPPVSKLDPEIYGPQESALKDEHILGHL---NGMTVQQALDEKKLFMV   500

                          α8 (466-475)        α9 (481-486)  β11 (491-494)
CsLOX15    KEWPLKSELDPTIYGPPESKITTELVEKFIKVYGYNNINEALKEKRLFML   493
CsLOX16    TEWPLKSELDPSIYGPQESKITTEIVEQQLKAYDYT-LLDARREKKLYLL   492
CsLOX17    TEWPLKSELDPSIYGPQESKITTEIVEQQLKAYDYT-LHDARREKKLYLL   492
CsLOX19    TEWPIKSKLDPVIYGPQESSITEEMINQEIG--GIMKVDEAIKQKKLFIL   490
CsLOX18    TEWPLKSKLDPNVYGSAESAITKEIIDKEIK--GFMTVEEAIEQKKLFML   497
CsLOX20    KEWPLKSKLDPEIYGPAESAITAELIEREIK--GFMTIDEAINEKKLFIL   284
CsLOX21    TEWPMKSKLDPEIYGPPESAITTELIEREIK--GFMTLDEAMREKKLFIL   474

Consensus  XXXDXXXPXXXXXXXXXXXXXYXXRXXXXXXXXXXLXPXXIELXXPXXXX   650
GmLOX1     DYHDIFMPYVRQINQLNSAKTYATRTILFLREDGTLKPVAIELSLPHSAG   457
GmLOX2     DYHDVFMPYIRRINQT-YAKAYATRTILFLRENGTLKPVAIELSLPHPAG   485
GmLOX3     GHHDPIMPYLRRINAT-STKAYATRTILFLKNDGTLRPLAIELSLPHPQG   476
GmVLXD     DYQDAFIPYLTRINSLPTAKAYATRTILFLKDDGTLKPLAIELSKPHPDG   483
GmVLXB     DHHDYLIPYLRKINAN-TTKTYATRTIFFLKNDGTLTPLAIELSKPHPQG   471
AtLOX1     DHHDTLMPYLGRVNTT-TTKTYASRTLLFLKDDGTLKPLVIELSLPHPNG   478
CsLOX1     NHHDMLFPYLRRINST-SSKIYATRTLLFLKNDGTLKPLAIELSLPHPDG   479
CsLOX2     DHHDTLIPFLRRINAT-NTKIYATRTIIFLQKDGTLKPLAIELSLPHPGG   574
CsLOX3     DHHDSLMPFLTLINST-DSKIYATRTLLLLQDDGTLKPLAIELSLPHPQG   488
CsLOX4     DHHDSFMPFVRRVNDTTSSKIYATRTLLFLTNDGTLKPLAIELSLPHPNG   479
CsLOX5     DLHDAFMPYLRRINTT-PTKTYATRTLLFLKDDGTLKPLAIELSLPHPKG   467
CsLOX6     DHHDSFMPYLRRINST-LTKTYASRTLLFLKDDGTLKPLAIELSLPHPNG   478
CsLOX7     DHHDSFMPYLTRINST-ATKTYASRTLLLLKNDGTLKPLAIELSLPHPKG   490
CsLOX8     DHHDSFIPYMRRINTT-LTKAYATRTILFLTKDGTLKPIAIELSLPHQDG   488
CsLOX9     DHHDSYIPYLRRINST-SSKAYATRTLVFLTSEGTLKPVAIELSLPHPNG   494
CsLOX10    DHHDSFIPYLRRINTT-PTKAYATRTLLFLANDGTLRPIAIELSLPHPDG   487
CsLOX11    DHHDSFIPYLRRINTT-PTKAYATRTLLFLANDGTLRPIAIELSLPHPDG   487
CsLOX12    DHHDTFMPYLKRINKT-STKAYATRTILFLTKEEKLMPVAIELSLPKDGP   328
CsLOX13    DYHDMLLPFIERMNSLPGRKAYASRTVFFYTKSGVLKPLVIELSLPPSSS   552
CsLOX14    DYHDIFLPFLDKINSLDGRRTYATRTIYFLSPQGTLKPVAIELSLPQTGP   550

           β11                    β12 (517-523)     β13 (529-537)
CsLOX15    DYHDVLLPYVSKVRELENKTLYGSRTLFFLTPYGTLLPLAIELTRPPMDG   543
CsLOX16    DYHDVLLPYVSKVRKLEKRTLYGSRTLFFLTPYGTLMPMAIELTRPPMDG   542
CsLOX17    DYHDVLLPYVSKVRKLEKRTLYGSRTLFFLTPYGTLMPMAIELTRPPMDG   542
CsLOX19    DYHDLLLPFVHQVRELKGTTLYGSRTVFFLNPDGTLRPLAIELTRPPHLD   540
CsLOX18    DYMDLLLPFVEKVRELEGTTLYGSRALFFLTEEGTLRPLAIELTRPQMDD   547
CsLOX20    DYHDLLLPYVKKVREIEGTTLYGSRTLFFLTLDGTLRPLAIELTRPPMDG   334
CsLOX21    DYHDLLLPYVKKVREIKGTTLYGSRTLFFLTLEGTLRPLAIELTRPPMDG   524

Consensus  XXXXXXXXXXXXXXXXXXXXXXXWXXXKXXXXXXDXXXHQXXXHWXXTH   700
GmLOX1     DLSAAVSQVVLPAKEGVES---TIWLLAKAYVIVNDSCYHQLMSHWLNTH   504
GmLOX2     DLSGAVSQVILPAKEGVES---TIWLLAKAYVVVNDSCYHQLMSHWLNTH   532
GmLOX3     DQSGAFSQVFLPADEGVES---SIWLLAKAYVVVNDSCYHQLVSHWLNTH   523
GmVLXD     DNLGPESIVVLPATEGVDS---TIWLLAKAHVIVNDSGYHQLVSHWLNTH   530
GmVLXB     EEYGPVSEVYVPSSEGVEA---YIWLLAKAYVVVNDACYHQIISHWLNTH   518
AtLOX1     DKFGAVSEVYTPG-EGVYD---SLWQLAKAFVGVNDSGNHQLISHWMQTH   524
CsLOX1     DQFGVVSKVFTPTKEGVES---HIWQLAKAYAAVNDSGVHQLISHWLNTH   526
CsLOX2     DHFGCISKVYTPADKGVDG---SLWQLAKAYVNVNDSGYHQLISHWLKTH   621
```

```
CsLOX3     YEHGAVSKVFTPEEKGIGE---TIWQLAKAYVAVNDSGYHQLISHWLNTH   535
CsLOX4     DALGAVSKICTPAEQGVES---TIWQLSKAYVTVNDYGHHQLVSHWLNTH   526
CsLOX5     DIYGSVSKVYTPAENGVEN---TIWQLAKAYVAVNDSGYHQLISHWLHTH   514
CsLOX6     DQYGAVSKVYTPSKEGVES---AIWELAKAYVAVNDSGYHQLISHWLNTH   525
CsLOX7     DQFGAVSKVYTPAEEGVGA---TIWQLAKAYAAVNDSGYHQLISHWLNTH   537
CsLOX8     DEHGAVSKVYTPTEEGVEG---TIWQLAKAYVAVNDVCYHQVYSHWLKTH   535
CsLOX9     DEFGVVSKVYTPAEEGVEG---TIWQLAKAYAAVTDSGYHQLISHWLNTH   541
CsLOX10    DEFGVVSKVYTPSEDGVDG---TIWQLAKAYAAVNDSGYHQLNSHWLNTH   534
CsLOX11    DEFGVVSKVYTPSEDGVDG---TIWQLAKAYAAVNDSGYHQLNSHWLNTH   534
CsLOX12    DENGADSTVFTPPCDNNDPHKYATWQLAKGYVAVNDSGYHQLYSHWLKTH   378
CsLOX13    SL-ESNKTVYTH---GHDATTHWIWKLAKAHVCSNDAGVHQLVNHWLRTH   598
CsLOX14    SS--RSKRVVTP---PVDATSNWVWQLAKAHVCTNDAGVHQLVNHWLRTH   595

           β14 (549-550)           α10 (560-586)
CsLOX15    KP-QW-KEVYTPM--NWHSTDLWLWRLAKAHVLAHDSGVHQLVSHWLRTH   589
CsLOX16    KP-QW-KEVYTPN-SNWHSTDIWLWKLAKAHVLAHDAGVHQLVSHWLRTH   589
CsLOX17    KP-QW-KEVYTPN-SNWHSTDIWLWKLAKAHVLAHDATVHQLVSHWLRTH   589
CsLOX19    SP-RW-NQVFSP---CWHSTGVWLWRLAKVHVLAHDSGYHQLVCHWLKTH   585
CsLOX18    KP-QW-KQVFTP---TWNATGDWLWRLAKAHVLAHDSGVHQLVSHWLRTH   592
CsLOX20    KP-QW-KEVFTP---SWHATAVWLWRFAKAHVLAHDSGYHQLVSHWLRTH   379
CsLOX21    KP-QW-KEVFSP---SWHATGVWLWRFAKAHALAHDSGYHQLVSHWLRTH   569

Consensus  XXXEPXXJXXXRXXSXXHPXXXLLXPHXRXXXXJNXXARXXLXXXXGXXE   750
GmLOX1     AAMEPFVIATHRHLSVLHPIYKLLTPHYRNNMNINALARQSLINANGIIE   554
GmLOX2     AVIEPFIIATNRHLSALHPIYKLLTPHYRDTMNINALARQSLINADGIIE   582
GmLOX3     AVVEPFIIATNRHLSVVHPIYKLLHPHYRDTMNINGLARLSLVNDGGVIE   573
GmVLXD     AVMEPFAIATNRHLSVLHPIYKLLYPHYRDTININGLARQSLINADGIIE   580
GmVLXB     AVVEPFVIATNRHLSVVHPIYKLLFPHYRDTMNINSLARKSLVNADGIIE   568
AtLOX1     ASIEPFVIATNRQLSVLHPVFKLLEPHFRDTMNINALARQILINGGGIFE   574
CsLOX1     AAIEPFVIATNRQLSVLHPIHKLLQPHFRDTMNINAFARQILINAGGILE   576
CsLOX2     AAIEPFVIATNRQLSVVHPINKLLHPHFRDTMNLNAVARQILINAGGALE   671
CsLOX3     AVIEPFIIATNRQLSVLHPIYKLLHPHFRDTMNINALARQTLINAGGILE   585
CsLOX4     AVAEPFVLATNRQLSVLHPIHKLLHPHFRDTMNINALARQLLVNGGGIIE   576
CsLOX5     AVIEPFVIVTNRQLSVVHPIYKLLQPHFRDTMNINSLARQALINADGPLE   564
CsLOX6     AVIEPFVIATNRQLSALHPIHKLLQPHFRDTMNINALARQTLINAGGILE   575
CsLOX7     AVIEPFVIATNRQLSVLHPIYKLLQPHYRDTMNINALARQTLINAEGILE   587
CsLOX8     CVSEPFVIATNRQLSVLHPIHKLLQPHFRDTMNINALARQTLINADGLVE   585
CsLOX9     AVIEPFVIATNRQLSALHPIYKLLQPHYRDTMNINALARQSLVSVDGVIE   591
CsLOX10    AVIEPFVIATNRQLSALHPIYKLLHPHYRDTMNINALARQSLVSADGIIE   584
CsLOX11    AVIEPFVIATNRQLSALHPIYKLLHPHYRDTMNINALARQSLVSADGIIE   584
CsLOX12    AVIEPFVIATNRQLSVIHPIYKLLHPHFRGTMSINARARTILVNADGTIE   428
CsLOX13    ACMEPYIIATHRQLSSMHPVYVLLHPHMRYTLEINALARQSLINGGGIIE   648
CsLOX14    ASLEPFILSAHRQMSAMHPIYKLLDPHMRYTLEINALARQNLINADGVIE   645

             α11 (592-602)        α12 (609-630)          β(638-639)
CsLOX15    CAVEPYIIATNRQLSAMHPIHRLLKPHFRYTMEINALARESLINAGGIIE   639
CsLOX16    CCVEPYVIATNRQLSAMHPINRLLQPHLRYTMEINALARNTLINAGGVIE   639
CsLOX17    CCVEPYVIATNRQLSAMHPINRLLQPHLRYTMEINALARNILISAGGIIE   639
CsLOX19    SITEPYVIATNRQLSIMHPVHRLLHPHFRYTMEINALAREALINADGTIE   635
CsLOX18    CVTEPYIIATKRQLSAMHPIFRLLHPHFRYTMEINALARQLLINSNGIIE   642
CsLOX20    CATEPYIIATNRQLSVMHPIYRLLHPHFRYTMEINALAREALVNAGGIIE   429
```

```
CsLOX21    CVTEPYIIAANRQLSVIHPIYRLLHPHFRYTMEINALAREALVNAGGIIE   619

Consensus  XXXXXXXXXXEXXXXXYXXXWXXXXXXXPXXLXXRXXXXXXXXXXXXXXX   800
GmLOX1     TTFLPSKYSVEMSSAVYK-NWVFTDQALPADLIKRGVAIKDPSTPHGVRL   603
GmLOX2     KSFLPSKHSVEMSSAVYK-NWVFTDQALPADLIKRGVAIKDPSAPHGLRL   631
GmLOX3     QTFLWGRYSVEMSAVVYK-DWVFTDQALPADLIKRGMAIEDPSCPHGIRL   622
GmVLXD     KSFLPGKYSIEMSSSVYK-NWVFTDQALPADLVKRGLAIEDPSAPHGLRL   629
GmVLXB     KTFLWGRYSLEMSAVIYK-DWVFTDQALPNDLVKRGVAVKDPSAPHGVRL   617
AtLOX1     ITVFPSKYAMEMSSFIYKNHWTFPDQALPAELKKRGMAVEDPEAPHGLRL   624
CsLOX1     FTVFPAKYAMEMSSAVYK-NWVFPEQALPEELMKRGMAVKDSSAPHGLRL   625
CsLOX2     ATVFPGRYCMEMTSASYK-EWTFPGQALPVDLIKRGVAVEDENSPHGLRL   720
CsLOX3     MTVFPGKYAMEMSAVIYK-NWVFTDHALPADLLKRGVAVEDSSGPHGLKL   634
CsLOX4     SVVFPGKYSMEISSAAYK-NWNLAEQAHPADLIKRGVAVEDPSSPHGVRL   625
CsLOX5     LTVFPRKYSMEMSAVIYK-DWVFPEQALPADLLKRGVAIKDPNSPHGLRL   613
CsLOX6     ITVFPGKYAMEMSAVVYK-NWIFPEQALPAELLKRGVAVEDPNSPHGLRL   624
CsLOX7     STVFPGKYALEMSAVVYK-NWVFTEQALPADLLKRGMAVKDPNSPHGLRL   636
CsLOX8     LAFFQGKYAMESSSLIYK-DWVFTEQALPTDLLKRGVAVKDENSPHGLRL   634
CsLOX9     STFLPGKYAMELSAVVYK-DWVFTDQALPADLLKRGIAEKDVNAPHGLRL   640
CsLOX10    STFFQGKFALESSAIIYK-DWKLTEQALPADLLKRGVAVKDQNSPHGVRL   633
CsLOX11    STFFQGQFALESSAIIYK-DWKFTEQALPADLLKRGVAVKDKNSPHGLRL   633
CsLOX12    SVFFQGKYAMESSSAVYKNNWAFNKQGLPADLIQRGLVDEKTKKPCEL--   476
CsLOX13    ASFSPGKYAMELSSAAYESQWRFDMEALPADLLRRGMAVEDPSMPCGVKL   698
CsLOX14    ACFTAGRYGMEISAAAYKSMWRFDQENLPADLVRRGMAVPDPTQPHGVKV   695

                     α13 (648-657)          α14 (669-672)
CsLOX15    TAFAPGKYSMELSSVMYDKQWRFDLQALPADLIHRRMAVEDKDSEHGVRV   689
CsLOX16    STFAPGKYCLELSSVIYDKQWRFDLQGLPADLVHRGMAVEDETCEHGLRL   689
CsLOX17    SIFAPGKYCLELSSVIYDKQWRFDLQGLPADLIHRGMAVEDETCEHGLRL   689
CsLOX19    TAFSPGKYSVELSSVAYDKEWRFDLQALPADLIDRGMAVEDPNSPHGLKL   685
CsLOX18    STFSPGKFSMEISSAAYDKLWRFDHEALPADLISRGIATEDPDSPHGLKL   692
CsLOX20    SSFSPGKYSMELCADAYDQIWRFDLEALPADLINRGMAVEDPSAPHGLKL   479
CsLOX21    SSFSPGKYANELCADAYDKQWRFDQQALPADLIKRGMAVEDSSAPHGLKL   669

Consensus  XXXBYPXXXDGLXXWXXXXXWVXXYXXXXYXXXXXXXXXDXEJQXXXXEX   850
GmLOX1     LIEDYPYAADGLEIWAAIKTWVQEYVPLYYAR-DDDVKNDSELQHWWKEA   652
GmLOX2     LIEDYPYAVDGLEIWAAIKTWVQEYVSLYYAR-DDDVKPDSELQQWWKEA   680
GmLOX3     VIEDYPYAVDGLEIWDAIKTWVHEYVFLYYKS-DDTLREDPELQACWKEL   671
GmVLXD     VIEDYPYAVDGLEIWDAIKTWVHEYVSLYYPT-DAAVQQDTELQAWWKEA   678
GmVLXB     LIEDYPYASDGLEIWDAIKSWVEEYVSFYYKS-DEELQKDPELQAWWKEL   666
AtLOX1     RIKDYPYAVDGLEVWYAIESWVRDYIFLFYKI-EEDIQTDTELQAWWKEV   673
CsLOX1     LIQDYPYGVDGLEIWYAIKTWVEDYCSFYYKT-DEAIQKDSELQSWWKEV   674
CsLOX2     LIDDYPFAVDGLEIWSAIKTWVKDYCSLYYKT-DKMVQEDYELQSWWKEL   769
CsLOX3     LIEDYPFAVDGLEIWSAIHTWVTEYCNIYYET-DKMVKEDTELQSWWTEV   683
CsLOX4     LIEDYPYAVDGLEIWAAMKNWVQEYCSFYYKS-DEIVQNDKELQAWWKEL   674
CsLOX5     LIEDYPYAVDGLEIWSAIHSWVEEYCSYYYKT-DDIIQKDSELQAWWKEL   662
CsLOX6     LIEDYPYAVDGLEIWSAIKSWVEEYCSYFYKT-DDIVQKDCELQAWWKEL   673
CsLOX7     LIEDYPFAADGLEIWSAIKSWVEEYCSFYYKA-DDTVQNDSELQAWWKEV   685
CsLOX8     LIEDYPYAVDGLEIWFAINSWVKEYCSYYYKT-DDTIQNDTEIQAWWKEI   683
CsLOX9     LIEDYPYAVDGLEIWSAIKAWVKEYCSHYYNS-DASVQRDTELQAWWKEV   689
CsLOX10    LIEDYPYAVDGLEIWSAIKTWVKEYCSFYYKT-DATVQKDTELQAWWKEV   682
CsLOX11    LIEDYPYAVDGLEIWSAINTWVKEYCSFYYKT-DATVQKDTELQAWWKEV   682
```

```
CsLOX12    -VKNYPYAVDGLEIWNAINKWVKEYCDIYYKGEDKKVQNDKEIQNWWKEI    525
CsLOX13    VIEDYPYAADGLLVWSAIKEWVESYVEHHYVE-PNSVRTDVELQAWWDEI    747
CsLOX14    VIEDYPYANDGLLIWGAIENWVSTYVNRYYPN-SSVVCNDLELQNWYSES    744

                      α15 (700-718)               α16 (728-740)
CsLOX15    IIEDYPYANDGLLIWSSIKQWVTDYVNHYYPI-SSEVERDEELQAWWTEI    738
CsLOX16    TIDDYPYANDGLLIWDAIKEWVTEYVKHYYAN-SSEVESDEELQEWWKEI    738
CsLOX17    TIEDYPYANDGLLIWDAIKEWVSEYINHYYAN-SSEIESDEELQEWWKEI    738
CsLOX19    TIEDYPYANDGLIMWDSIKEWVTDYINHYYPK-ASLVESDQELQAWWTEI    734
CsLOX18    NIEDYPFANDGLLIWDALKQWVTSYVNHYYSD-SSQVELDEELQSWWTEI    741
CsLOX20    TIEDYPFANDGLVLWDIIKQWVTDYVNYYYPE-PTLIESDLELQSWWTEI    528
CsLOX21    TIEDYPFANDGLALWDIIKQWVTDYVNYYYPE-QTLIESDLELQSWWTEI    718

Consensus  XXXGHXDXXXXXXWXXXXTXXXLXXXXXXXXWXXSXXHAXXNFGQYXXXX    900
GmLOX1     VEKGHGDLKDKPWWPKLQTLEDLVEVCLIIIWIASALHAAVNFGQYPYGG    702
GmLOX2     VEKGHGDLKDKPWWPKLQTIEELVEICTIIIWTASALHAAVNFGQYPYGG    730
GmLOX3     VEVGHGDKKNEPWWPKMQTREELVEACAIIIWTASALHAAVNFGQYPYGG    721
GmVLXD     VEKGHGDLKEKPWWPKMQTTEDLIQSCSIIVWTASALHAAVNFGQYPYGG    728
GmVLXB     VEVGHGDLKDKPWWQKMQTREELVEASATLIWIASALHAAVNFGQYPYGG    716
AtLOX1     REEGHGDKKSEPWWPKMQTREELVESCTIIIWVASALHAAVNFGQYPVAG    723
CsLOX1     REEGHGDKKNEPWWPKMQTRQELIDSCTIIIWIASALHAAVNFGQYPYAG    724
CsLOX2     REEGHGDKKDEPWWPEMQTREDLIETCTIIIWIASALHAAINFGQYPYGG    819
CsLOX3     RIEGHGDKKKEPWWPKMETISELINSCTIIIWIASALHAAVNFGQYPYAG    733
CsLOX4     REVGHGDLKDKSWWPSMQTREELVETCTTAIWTTSALHAAVNFGQYPYGG    724
CsLOX5     VEVGHGDKKNEPWWPKMQTRGELVESCATVIWIASALHAAVNFGQYPYAG    712
CsLOX6     REVGHGDKKDELWWNKMQTREELIDSCTIMIWIASALHAAVNFGQYPYAG    723
CsLOX7     RDVGHGDKKDESWWPKMLTREELVESCTIIIWISSALHAAVNFGQYPYAG    735
CsLOX8     REVGHGDKKDETWWPKMQTIEELVESCTTIIWISSALHAAVNFGQYAYEG    733
CsLOX9     REVGHGDKKDETWWPKMKTREELVESCTILIWISSALHAAVNFGQYSFTG    739
CsLOX10    REVGHGDKKHEAWWPKMQTREELVESCTTLIWISSALHAAVNFGQYSFTG    732
CsLOX11    REVGHGDKKHEAWWPKMQTREELVESCTTLIWISSALHAAVNFGQYSFTG    732
CsLOX12    KEVGHGDKKEYDGWPEMKTFNELVESCTIIIWISSALHAAVNFGQYSFAA    575
CsLOX13    KNKGHYDKRNEPWWPKLETKQDLSGVLTTIIWIASGQHAAINFGQYPFGG    797
CsLOX14    INVGHADVRNASWWPKLETPEDLTSILTTLIWLASAQHAALNFGQYPYGG    794

           α16                      α17 (758-780)
CsLOX15    RTVGHADKKDAPGWPDLKTKQDLIDIVTNMAWTASAHHAAVNFGQYAYAG    788
CsLOX16    RTVGHADKKE--GWPDLKTREDLIDIVTNIAWIASGHHAAVNFGQYAFSA    786
CsLOX17    RTVGHADKKE--GWPDLKTREDLIDIVTNIAWIASGHHAAVNFGQYAFAA    786
CsLOX19    RTVGHADKKDEPWWPVLNTPKDLIEILTTMLWVTSGYHAAVNFGQYTYGG    784
CsLOX18    RTVGHEDKKDEPWWPTLETPEDLIQILTTIVWVTSGHHAAVNFGQYTYAG    791
CsLOX20    RTVGHGDKKDEPWWPILKTPNDLVQIITTIVWVTSGHHAAVNFGQYTYAG    578
CsLOX21    RTVGHGDKKDEPWWPLLKTPNDLIGIVTTIVWVTSGHHASVNFGQYTYAG    768

Consensus  XXXNRXXXXRXXXPXXXXXXXXXXXXXXXXXXXXXXXXXXXXXXXXXXXX    950
GmLOX1     LIMNRPTASRRLLPEKG----TPEYEEMINNHEKAYLRTITSKLPTLISL    748
GmLOX2     FILNRPTSSRRLLPEKG----TPEYEEMVKSHQKAYLRTITSKFQTLVDL    776
GmLOX3     LILNRPTLSRRFMPEKG----SAEYEELRKNPQKAYLKTITPKFQTLIDL    767
GmVLXD     LILNRPTLARRFIPAEG----TPEYDEMVKNPQKAYLRTITPKFETLIDL    774
GmVLXB     LILNRPTISRRFMPEKG----SPEYDALAKNPEKEFLKTITGKKETLIDL    762
AtLOX1     YLPNRPTISRQYMPKEN----TPEFEELEKNPDKVFLKTITAQLQTLLGI    769
```

```
CsLOX1     YLPNRPTLSRRFMPEKG----TPEYEELQSNPEKAFLKTITAQLPTLIGV   770
CsLOX2     FPPNRPSMSRRFIPEEG----TPEYNELKTDPEKALLKTITGQLLSVLGI   865
CsLOX3     YLPNRPTVSRRFMPEPG----TPEYKELEMYPDVAYLKTITAQFQTLLGV   779
CsLOX4     YIPNRPTISRRLMPEEG----TREYEELKTNPEKAFLRTITSELQSLVEI   770
CsLOX5     YSPNRPTVSRQFMPVQG----TAEYELLRTDPEKGFLETITAELQTLIGI   758
CsLOX6     FLPNRPTLSRRFMPEKG----TPEYEELQSDPEKGFLKTITPLLQTLIGV   769
CsLOX7     YLPNRPTVSRRFMPEEG----TPEYEQLKSDPEKGFLLTITSEFQTLIGV   781
CsLOX8     FLLNRPTLSREFMPEKG----TPKYEELKSDPEKGFLSIITPEFQSLLGI   779
CsLOX9     YLPNRPTLSRRFMPEEG----TPEYEQLKSDPEKGFLLTITSEFQSLVGI   785
CsLOX10    YLPNRPTLSRRFMPEEG----TPEYEQLKSDPEKGFLLTITPEFQSLIGI   778
CsLOX11    YLPNRPTLSRRFMPEEG----TPEYEQLKSDPEKGFLLTITPEFQSLIGI   778
CsLOX12    FYPNRPTLSRQFMPMGG----TT---LGVDDEESYFLSTFTSKKQSLEMI   618
CsLOX13    YVPNRPTLLRKLIPHED----DTDYEKFMLNPQRTFLSSLPTQLQATKVM   843
CsLOX14    YVPNRPPLMRRLIPEEN----DPEYATFVADPQKYFLLALPSVLQSTKFM   840

                                                 α18 (820-827)    α19 (831-844)
CsLOX15    YFPNRPTITRTVMPSEEKEYNLDAWKHFKNSPEDALLKCLPTQLQAGLVV   838
CsLOX16    YFPNRPSLTRTNMPSEEKESKPEVWKAFKANPEDTILKCFPSQLQAAKNM   836
CsLOX17    YFPNRPPVTRTNMPSEEKESKPEVWKAFKANPEDTILKCFPSQREAALNM   836
CsLOX19    YFPNRPTIARTKMPTEDPD--EEEWKSFVKKPESALLKCFPSQLQATRVM   832
CsLOX18    YFPNRATIARTNVPTEDPT--DEEWKYFIDKPEGTLLQCLPSKLQAAKVM   839
CsLOX20    YFPNRPTIARINVPTEDAS--DEFLEKFWAKPEEALMQCFPSQIQATTVM   626
CsLOX21    YFPNRPTIARTNMPTEDAS--EEVLEKFWAKPEEALMQCFPSQIQATTVM   816

Consensus  XXXXXLSXHXXXEXYJGXXXXXXXXWXXXXXXXXXFXXXXXXXXXJXXXX  1000
GmLOX1     SVIEILSTHASDEVYLGQRDN--PHWTSDSKALQAFQKFGNKLKEIEEKL   796
GmLOX2     SVIEILSRHASDEVYLGQRDN--PHWTSDSKALQAFQKFGNKLKEIEEKL   824
GmLOX3     SVIEILSRHASDEVYLGERDN--PNWTSDTRALEAFKRFGNKLAQIENKL   815
GmVLXD     SVIEILSRHASDEIYLGERET--PNWTTDKKALEAFKRFGSKLTGIEGKI   822
GmVLXB     TIIEILSRHASDEFYLGQRDG-GDYWTSDAGPLEAFKRFGKNLEEIEKKL   811
AtLOX1     SLIEILSTHSSDEVYLGQRDS--KEWAAEKEALEAFEKFGEKVKEIEKNI   817
CsLOX1     ALIEILSRHASDEVYLGQRDT--PQWTSDTAPLEAFDRFGKKLAEIEEKI   818
CsLOX2     SLVEILSRHSSDEVYLGERDT--PEWTTDKEAEKAFEKFGNKLREIELKI   913
CsLOX3     SLIEVLSRHTGDEIYLGQRDT--MKWTMDEEALKAFTRFGDRLREIENRI   827
CsLOX4     SIIEVLSKHASDEVYLGERE---EKWTAEAEPLQAFERFGRRLSEIEDGI   817
CsLOX5     SLIEILSRHTSDEVYLGQTDT--PEWTAEVDTLQAFERFGKKLDVIGNRI   806
CsLOX6     SLIEVLSRHTSDEVYLGQRDT--PEWTSEGHSLQAFERFGKKLIQIEDNI   817
CsLOX7     SLIEILSRHASDEVYLGQRDY--DDWTAEAGPLQAFERFGRKLAQIEDKM   829
CsLOX8     SLVEILSRHASDEIYLGQREN--PDWTSESDPLQAFERFGKKLSEIEDKI   827
CsLOX9     ALVEVLSRHASDEVYLGQREN--PDWTLDAGPLQAFDKFGRKLVQIEDAI   833
CsLOX10    SLVEILSRHASDELYLGQREN--PDWTAEAEPLNAFERFGTKLAQIEDRI   826
CsLOX11    SLVEILSRHASDELYLGQREN--PDWTAEAEPLNAFERFGTKLAQIEDRI   826
CsLOX12    YLIAQLSSHESDEVYLGKNED--VDWTSDKKALDAFKKFQTQLRAIEDDI   666
CsLOX13    AVQDTLSTHSADEEYLGQVNPLHTHWTNDQIILKIFSKYSAQLEEIDKII   893
CsLOX14    AVVDTLSTHSPDEEYIGERQQ-PSIWSGDPEIVEAFYEFSAQVKNIEKEI   889

             α19                         α20 (866-889)
CsLOX15    AVLDVLSTHSPDEEYLGDKM--EPSWGSNLVIAEAFNRFNKRMNEIESII   886
CsLOX16    AALDLLSTHASDEEYLGHHV--ESAWKEDSKILDSFKKFQQRIKQIEDII   884
CsLOX17    LALDLLSTHASDEEYLGHHV--ESAWKDDSKIFESFKKFQQRIKKIEDII   884
CsLOX19    AVLDILSNHSPDEEYLGEKM--EKAWADDPVIKAAFERFNGRLKVLEGII   880
```

```
CsLOX18    AVLNVLSTHSPEEEYLGEAL--EPSWGDDTYIKATFEQFSGRLKEIEGII   887
CsLOX20    AVLDILSNHSPDEEYLGEKI--EPSWEEDDVIKAAFERFQGRLKELEGII   674
CsLOX21    AVLDILSNHSPDEEYLGENI--EPSWEENDVIKAAFERFNGRLKELEGII   864

Consensus  XXXNXBXXXXXNRXGXXXXXYXXLXXXXXXXXXXGXXXXGXPXSXSI    1047
GmLOX1     VRRNNDPSLQGNRLGPVQLPYTLLYPSSE----EGLTFRGIPNSISI     839
GmLOX2     ARKNNDQSL-SNRLGPVQLPYTLLHPNS-----EGLTCRGIPNSISI     865
GmLOX3     SERNNDEKL-RNRCGPVQMPYTLLLPSSK----EGLTFRGIPNSISI     857
GmVLXD     NARNSDPSL-RNRTGPVQLPYTLLHRSSE----EGLTFKGIPNSISI     864
GmVLXB     IEKNNDETL-RNRYGPAKMPYTLLYPSSE----EGLTFRGIPNSISI     853
AtLOX1     DERNDDETL-KNRTGLVKMPYTLLFPSSE----GGVTGRGIPNSVSI     859
CsLOX1     MNMNNDEKL-KNRVGPVKVPYTLLYPTSD----EGLTGKGIPNSVSI     860
CsLOX2     EKMNKDQKL-KNRVGPVKMPYTLLYPSSE----GGLTGKGIPNSVSI     955
CsLOX3     TEMNSNGKW-KNRVGPVKLPYTLLYPNTSGHPNEGLTGKGIPNSISI     873
CsLOX4     VKRNNDATL-KNRYGLVKFPYNLLIPNGE----IGIAAKGIPNSISI     859
CsLOX5     MSRNTDPNL-KNRVGPVKFPYTLLYPTSG----KGLTSKGIPNSVSI     848
CsLOX6     MRRNNDVNL-KNRVGPIKFPYTLLHPTSE----KGVTAMGIPNSVSI     859
CsLOX7     MSKNKDTNL-KNRAGPVNFPYTLLHPNGE----EGLSAKGIPNSISI     871
CsLOX8     TSRNKDRNM-KNRVGPISFPYTLLMPNGE----EGLSAKGIPNSISI     869
CsLOX9     VRRNNDLNM-KNRAGPVKFPYGLLIPTSD----QGLTGRGIPNSISI     875
CsLOX10    ASRNKDTNM-KNRAGPVNFEYGLLMPSSE----QGLTARGIPNSISI     868
CsLOX11    ASRNKDTNM-KNRAGPVNFEYGLLMPSSE----QGLTARGIPNSISI     868
CsLOX12    TTKNQNHE--KNRCGPIQFPYTLLFPTSE----PGITAKGIPNSISI     707
CsLOX13    NKRNKDNRL-KNRSGAGVPPYELLLPTSG----PGVTGRGIPNSISI     935
CsLOX14    ERRNNDPSL-RNRCGAGVLPYELLAPSSE----PGVTCRGVPNSVSI     931

           α20
CsLOX15    NEKNDNENL-RNRHGAGILSYELLKPFSE----PGVTNKGIPYSISI     928
CsLOX16    DERNDDENL-KNRHGAGILPYEFLKPFSE----EGITNKGVPYSISI     926
CsLOX17    DERNDDENL-KNRHGAGILPYEFLKPFSE----KGITNKGVPYSISI     926
CsLOX19    DEKNENPNL-RNRNGAGIIPYELLKPFST----PGVTGKGVPYSISI     922
CsLOX18    DERNGNQEL-RNRNGAGIAPYELLKPSSE----PGVTGMGVPYSISI     929
CsLOX20    DGRNTNKDL-KNRCGVGVVPYQLLKPFSE----PGVTSKGVPYSISI     716
CsLOX21    DERNTNKNL-RNRCGVGVVPYQLLKPFSE----PGVTSKGVPYSISI     906
```

Supplementary Figure S1: Amino acid sequence aligment of *Cannabis sativa* lipoxygenase (CsLOX) with soybean (*Glycine max)* wild type GmLOX1, GmLOX3, GmVLXB, GmVLXD, *Arabidopsis thaliana* AtLOX1, and. Conserved residues are highlighted in ash. Predicted secondary structural elements of CsLOX15 are highlighted in colours, β-sheets-blue, a-helix-green above the corresponding sequence. Accession numbers and uniport IDs are provided in supplementary table1.

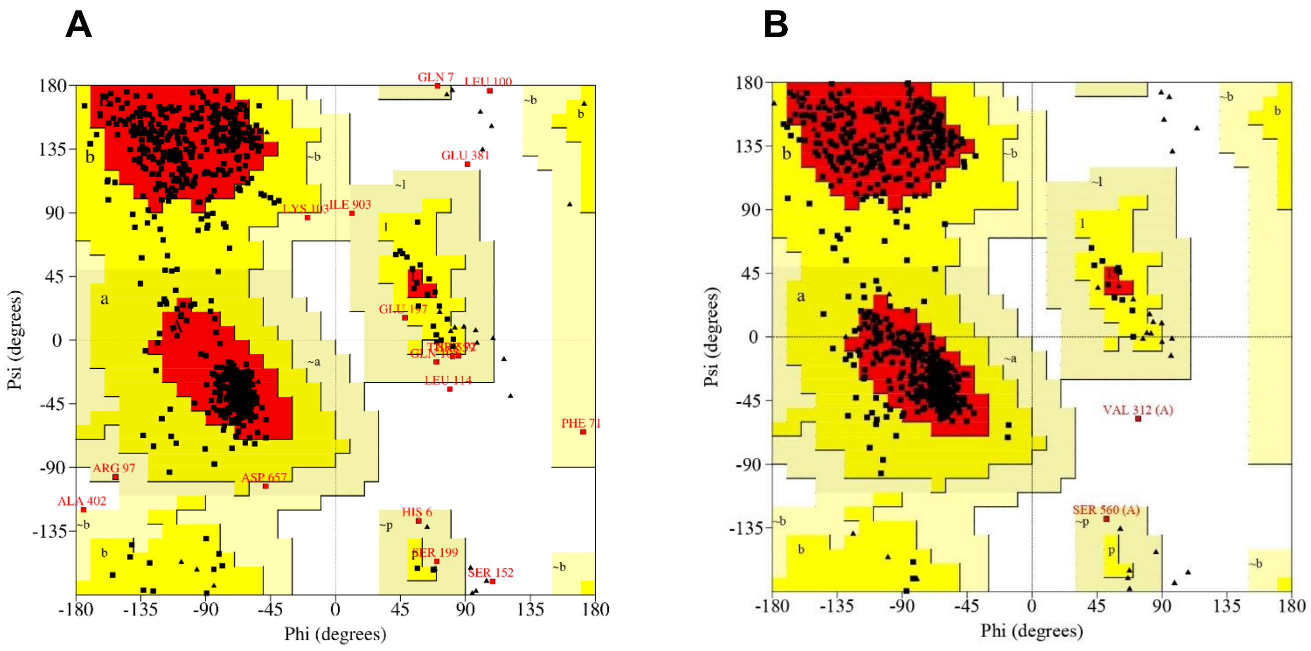

A – Plot statistics

Residues in most favoured regions [A,B,L]	709	86.1%
Residues in additional allowed regions [a,b,l,p]	97	11.8%
Residues in generously allowed regions [~a,~b,~l,~p]	12	1.5%
Residues in disallowed regions	5	0.6%
	----	------
Number of non-glycine and non-proline residues	823	100.0%
Number of end-residues (excl. Gly and Pro)	2	
Number of glycine residues (shown as triangles)	42	
Number of proline residues	61	

Total number of residues	928	

B – Plot statistics

Residues in most favoured regions [A,B,L]	639	89.7%
Residues in additional allowed regions [a,b,l,p]	71	10.0%
Residues in generously allowed regions [~a,~b,~l,~p]	1	0.1%
Residues in disallowed regions	1	0.1%
	----	------
Number of non-glycine and non-proline residues	712	100.0%
Number of end-residues (excl. Gly and Pro)	5	
Number of glycine residues (shown as triangles)	51	
Number of proline residues	50	

Total number of residues	818	

Supplementary Figure S2: Ramachandran plots for **(A)** CsLOX15 protein model and **(B)** GmLOX1. The putative structure of CsLOX15 obtained from Phyre2 (Kelley et al., 2015) was analysed using PROCHECK in SAVES v6.1[1].

[1] https://saves.mbi.ucla.edu/
